# Can dual-task high-velocity exercise training improve cognitive function in older adults? Secondary analysis of an 18-month cluster randomized controlled trial

**DOI:** 10.1093/ageing/afaf385

**Published:** 2026-01-23

**Authors:** Jamie L Tait, Rachel L Duckham, Timo Rantalainen, Catherine M Milte, Luana C Main, Caryl A Nowson, Kerrie M Sanders, Dennis R Taaffe, Keith D Hill, Gavin Abbott, Robin M Daly

**Affiliations:** Institute for Physical Activity and Nutrition (IPAN), School of Exercise and Nutrition Sciences, Deakin University, 1 Gheringhap St, Geelong, Victoria, 3220, Australia; Institute for Physical Activity and Nutrition (IPAN), School of Exercise and Nutrition Sciences, Deakin University, 1 Gheringhap St, Geelong, Victoria, 3220, Australia; Australian Institute for Musculoskeletal Science (AIMSS), Department of Medicine-Western Health, The University of Melbourne, Level 3 & 4, 176 Furlong Road, St Albans, Victoria, 3021, Australia; Department of Medicine, The Royal Melbourne Hospital, The University of Melbourne, Royal Parade, Parkville, Victoria, 3050, Australia; Finnish Institute of High Performance Sport KIHU, Rautpohjankatu 6, FI-40700, Jyväskylä, Finland; Institute for Physical Activity and Nutrition (IPAN), School of Exercise and Nutrition Sciences, Deakin University, 1 Gheringhap St, Geelong, Victoria, 3220, Australia; Institute for Physical Activity and Nutrition (IPAN), School of Exercise and Nutrition Sciences, Deakin University, 1 Gheringhap St, Geelong, Victoria, 3220, Australia; Institute for Physical Activity and Nutrition (IPAN), School of Exercise and Nutrition Sciences, Deakin University, 1 Gheringhap St, Geelong, Victoria, 3220, Australia; Department of Medicine, Western Health, The University of Melbourne, Level 3, 176 Furlong Road, St Albans, Victoria, 3021, Australia; Exercise Medicine Research Institute and School of Medical and Health Sciences, Edith Cowan University, 270 Joondalup Drive, Joondalup, Western Australia, 6027, Australia; Rehabilitation Ageing and Independent Living (RAIL) Research Centre, Monash University, 47–49 Moorooduc Highway, Frankston, Victoria, 3199, Australia; Institute for Physical Activity and Nutrition (IPAN), School of Exercise and Nutrition Sciences, Deakin University, 1 Gheringhap St, Geelong, Victoria, 3220, Australia; Institute for Physical Activity and Nutrition (IPAN), School of Exercise and Nutrition Sciences, Deakin University, 1 Gheringhap St, Geelong, Victoria, 3220, Australia

**Keywords:** dual-task training, power training, physical activity, ageing

## Abstract

**Background:**

Identifying strategies to mitigate age-associated cognitive decline is crucial. High-velocity power training enhances physical function in older adults and cognitive training has mixed cognitive benefits, however the combined effects of these interventions remain uncertain.

**Objective:**

This 18-month cluster randomized controlled trial investigated whether dual-task functional power training (DT-FPT) enhances cognition in older adults and assessed if responses differ by apolipoprotein-E and brain-derived neurotrophic factor (BDNF) polymorphisms.

**Subjects and Methods:**

Twenty-two independent-living retirement communities (300 residents, ≥65y at increased falls risk) were randomized to 12-months of group-based DT-FPT (6-months supervised +6-months maintenance, 45–60 minutes, 2/week) performed simultaneously with cognitive and/or motor tasks, followed by 6-months follow-up, or usual care control (CON). Cognitive domains were assessed using CogState at baseline, 6, 12 and 18-months. Z-scores were created to form composites for psychomotor-attention, learning-working memory and global cognition. *BDNF* and *APOE* polymorphism data were obtained from blood samples.

**Results:**

Overall, 223 (74%) participants completed the 18-month intervention; mean exercise adherence was 50% at 6-months and 40% at 12-months. Net benefits in choice reaction time and attention (0.17 SD, P = 0.016), psychomotor-attention (0.19 SD, P = 0.029), and a composite of psychomotor-attention, learning-working memory (0.11 SD, P = 0.046) were detected in DT-FPT vs CON after the 6-month supervised phase. At 12 and 18 months, benefits from DT-FPT relative to CON were extended to visual learning (0.29 SD, P = 0.013; 0.27 SD, P = 0.008) and learning-working memory (0.13 SD, P = 0.047; 0.18 SD, P = 0.013). CON exhibited a 0.19 SD net benefit for executive function (P = 0.003) after 18 months. BDNF Met carriers at 18 months showed improved working memory (0.35 SD, P = 0.042) and learning-working memory (0.37 SD, P = 0.011) in DT-FPT versus CON.

**Conclusions:**

In older retirement living residents, DT-FPT may improve cognitive domains critical for functional independence, with genotype potentially influencing these outcomes.

Australian New Zealand Clinical Trials Registry (ACTRN12613001161718). This project was funded by the National Health and Medical Research Council (NHMRC) (APP1046267).

## Key Points

- DT-FPT enhanced reaction time, attention, and aspects of memory in older adults.
- Benefits for visual learning and working memory emerged after 12 months of DT-FPT.
- Genotype may influence DT-FPT effects, based on findings in BDNF Met-carriers.

## Background

Age-associated cognitive decline and dementia are significant public health concerns without cure. Regular exercise including aerobic training, progressive resistance training (PRT) or the combination of both can benefit cognition in older adults [1], but questions remain about the optimal type and dose (frequency, intensity, duration) of training. Cognitive training involving the repetition of mental exercises (e.g. remembering word lists, locating visual information) is also advocated for cognitive improvement in older adults, although intervention findings are mixed [2]. Several systematic reviews and meta-analyses have reported that combined exercise and cognitive training (dual-task training) may be more effective for improving cognition than exercise alone [3, 4]. Furthermore, simultaneous exercise-cognitive training, whereby exercise is performed concurrently with a secondary cognitive and/or motor task, has been shown to be more effective for enhancing dual-task performance and cognition in older adults than sequential training where exercise and cognitive/motor tasks are performed separately [5], making it more clinically relevant for maintaining multitasking abilities than sequential training [6, 7].

Despite the reported benefits of simultaneous exercise-cognitive training, the optimal type(s) of exercise training for cognitive benefits remain unknown, with studies incorporating a variety of modalities including aerobic training, PRT, functional (stepping) training, or the combination, and exergaming [8]. High velocity-PRT, or power training, and challenging balance training are effective fall prevention strategies in older adults [9], and their combined application may enhance both neuromuscular and cognitive capacities critical for reducing fall risk, while also offering potential benefits to broader aspects of cognitive function. In particular, power training has been demonstrated to be effective for improving lower limb muscle power and functional performance in older adults [10], and can also enhance cognitive abilities (i.e. global cognition, working memory and processing speed) in frail [11], and cognitively impaired older adults [12]. Power training integrated within dual-task exercise may enhance cognition by engaging overlapping neural networks involved in motor control and executive function, including the prefrontal cortex, fronto-parietal networks, and cortico-striatal circuits among others [13, 14], strengthening connectivity between motor and cognitive regions. Challenging balance training including dual-task activities, when integrated within multimodal exercise-cognitive programs can also enhance cognitive function [8]. However, no studies to our knowledge have evaluated the impact of combining high-velocity power and balance training with functional mobility tasks (e.g. stepping, squatting) performed rapidly in a multicomponent exercise program, along with simultaneous cognitive training and motor tasks in older adults, to determine benefits for cognitive function.

Given that exercise is a modifiable lifestyle factor with cognitive benefits, it is important to consider whether genetic variation moderates these effects. Cognition may be influenced by genetic factors such as the possession of a Met allele on the gene encoding brain-derived neurotrophic factor (BDNF), which is associated with poorer cognition [15], and the *APOE*-ε4 variant coding for the apolipoprotein E protein, an important predictor for the development of Alzheimer’s disease and poorer cognitive performance in older adults [16]. Despite this, there is limited and inconclusive data on whether these polymorphisms moderate the effect of exercise on cognitive performance in older adults [17, 18], which may underlie potential effects of a dual-task exercise intervention. Therefore, the aim of this study, which represents a secondary analysis of an 18-month cluster randomized controlled trial (RCT) in which falls were the primary outcome, was to examine whether the 12-month dual-task functional power training (DT-FPT) program could improve cognition, compared to usual care, in older adults at increased risk for falls living independently in retirement villages. Secondary aims were to determine if there were any longer-term residual effects after 18 months, and whether the cognitive responses to DT-FPT varied by apolipoprotein-E (*APOE*) and *BDNF* polymorphisms.

## Methods

### Study design

This study is a secondary analysis of an 18-month cluster RCT incorporating a 12-month intervention with a 6-month follow-up that was conducted in 22 independent-living retirement communities with older adults at increased risk for falls. Participants were randomized by village to either: 1) a 12-month multicomponent, functional dual-task power training program with challenging balance/mobility training that was performed simultaneously with a secondary attention-demanding motor or cognitive task (DT-FPT, n = 11 villages, n = 156 participants), or 2) a usual care control group (CON; n = 11 villages, n = 144 participants). The study was divided into three 6-month phases (phase 1: supervised/structured exercise training; phase 2: a maintenance exercise training period; phase 3: follow-up) [19]. This study reports on the secondary outcomes, which included the evaluation of cognitive function over the 12-month intervention period and 6-month follow-up. Recruitment occurred over 14 months in three cohorts (February 2014 to April 2015) [20], with baseline and follow-up testing at 6, 12 and 18 months, conducted at each retirement village. To be eligible, villages needed a suitable venue and availability to host functional testing and twice-weekly exercise sessions (if randomized to intervention), independently living residents, and at least five eligible and interested participants. Cohort 1 included eight retirement villages (n = 82 participants), cohort 2 had six villages (n = 104 participants), and cohort 3 comprised eight villages (n = 114 participants) [20]. Cluster randomization by village was employed to reduce intervention contamination within each village site. Each village was given an ID number and group assignment by blocks of two, and randomization was stratified by village size (<75 or ≥ 75 residents) using computer-generated random numbers (Microsoft Excel), by a researcher not involved in the study. Testing personnel were blinded to group allocation, except for one researcher handling participant communication and administration of the cognitive tests. The study was approved by the Deakin University Human Research Ethics Committee (EC 2013–051) and registered on the Australian and New Zealand Clinical Trials Registry (ACTRN12613001161718).

### Participants

A total of 300 older adults (81 men and 219 women) from 22 retirement villages in greater metropolitan Melbourne and regional Victoria, Australia were recruited. Recruitment and screening details are previously published [19, 21]. Briefly, interested participants were screened and included if aged ≥65 years, scored ≥3 on a defined risk of falls algorithm adapted from prior research [22], scored ≤2 errors on the Short Portable Mental Status Questionnaire [23] to exclude cognitive impairment, spoke English proficiently and could walk unaided or with minimal assistance for at least 50 metres. Eligible participants were further screened using the Exercise and Sports Science Australia exercise-screening tool [24], with those answering ‘Yes’ to any questions required to obtain medical clearance before starting the intervention. Participants were excluded if they: 1) engaged in ≥150 minutes/week of moderate-to-vigorous physical activity or > 1 weekly session of a structured PRT or balance program over the past 3 months; 2) had musculoskeletal, neurological, acute, terminal, or unstable cardiovascular/respiratory conditions; 3) experienced a limb fracture within the past 3 months; or 4) had uncorrected visual impairment. Written informed consent was obtained by participants prior to participation.

**Intervention.**

Villages allocated to the exercise intervention were prescribed two 45 – 60 minute supervised, group-based (8 – 10 per group) sessions per week on non-consecutive days for 6 months. Sessions were led by trained exercise professionals, on-site at each village, who had completed a ‘train-the-trainer’ session to ensure consistent program delivery across villages. A detailed overview of each training session has been outlined previously [21]. Briefly, each training session involved four components: 1) a warm-up with rhythmic and range of motion exercises (e.g. marching); 2) 2 – 3 challenging balance and mobility exercises that simulated common daily functional tasks (e.g. multidirectional weight shifts, walking over obstacle courses); 3) high-velocity functional power training (FPT) including 5 – 6 lower limb exercises (e.g. squats, lunges) and at least one upper limb exercise (e.g. bent-over rows), with additional core stability and postural exercises (e.g. fit ball sit-ups); and 4) cool-down. Participants progressed through stepping exercises (2 sets of 10 – 20 repetitions, up to 50 repetitions) and FPT (2 sets of 10 – 15 reps) with a target of 4 to 6 (moderate to hard) on the 10-point Borg Rating of Perceived Exertion scale [25]. Dual-task training was performed simultaneously with the challenging balance/mobility and FPT exercises, involving a combination of cognitive (e.g. anagrams), visual (e.g. memory recall) and motor tasks (e.g. throwing a weighted ball). The program included a 2-week familiarization phase and three 8-week blocks [19].

**Control group.**

Participants in the usual care control group continued their normal everyday activities and received standard care from healthcare providers and community services. Participants were given a falls prevention booklet (Department of Health: ‘Don’t fall for it – falls can be prevented’, and ‘An active way to better health’) and encouraged to meet the recommended 150 minutes of weekly physical activity, with additional support through pamphlets and initial contact with the research team.

**Step-down maintenance exercise program and follow-up.**

Following 6 months of supervised training, the DT-FPT group entered a 6-month ‘step-down’ maintenance phase involving less supervised exercise support [21]. Village managers opted to continue with one of our trainers to deliver a once weekly (45–60 minute) supervised exercise class funded by the research team. Villages could also arrange an additional exercise session per week with the trainer, funded by the village and/or residents. Only one village took up this option for a second session, while three others completed a self-directed, unsupervised second session per week. All participants were also encouraged to complete one additional brisk walking session per week. After this phase, participants were monitored for an additional 6 months with no further training support or advice provided.

**Cognitive measures.**

Cognitive function was assessed at each village at baseline, 6 months, 12 months and 18 months, using the valid and sensitive CogState computerized cognitive battery (CogState Ltd, Melbourne, Australia). CogState has demonstrated minimal practice effects across repeated assessments, with prior studies reporting high test–retest reliability and negligible performance changes attributable to practice, supporting its suitability for repeated use in clinical and research settings [26]. The cognitive battery included the following tests: Groton Maze Learning Test (GMT; executive function, memory, and visuospatial learning); Detection task (DET; simple reaction time and processing speed); Identification task (IDN; choice reaction time and visual attention); One Card Learning task (OCL; visual learning); and One Back task (ONB; working memory) [27]. The battery was administered on a laptop, with standardized instructions and practice tests provided before each task. Outcome measures included reaction time, proportion of correct responses, and total number of errors. As previously reported [28], reaction time scores of IDN, DET and ONB were log_10_ transformed, while the square root of the proportion of correct responses on the OCL task was arcsine transformed. Individual test scores were transformed into z-scores based on the baseline mean and standard deviation (SD) of the total sample, and composite scores were created: global cognitive function (GCF: average for GMT, DET, IDN, OCL, and ONB), Learning-Working memory (L-WM: average for OCL and ONB), Psychomotor function-Attention (Psy-Att: average for DET and IDN), and the CogState Brief Battery (CBB: average for DET, IDN, OCL and ONB). Higher scores on these composites indicate better performance [29]. Participants with z-scores ≤ −1.0 SD on three or more tests were classified as having mild cognitive impairment [MCI; based on communication with CogState Pty Ltd and [29].

### Genotyping

Genotype-specific cognitive and biomarker responses were assessed using *BDNF* and *APOE* polymorphism data from venous blood samples. Single nucleotide polymorphisms were genotyped as described by the manufacturer (Agena Bioscience, San Diego, USA) for BDNF (rs6265), ApoE-ε3 (rs7412) and ApoE-ε4 (rs429358) using matrix-assisted laser desorption/ionization time-of-flight mass spectroscopy (MALDI-TOF MS) (Agena Bioscience, San Diego, USA). Participants were classified based on *APOE* gene status: presence of at least one ε4 allele or none, and *BDNF* genotype: at least one Met allele or Val/Val homozygotes. Analyses included only participants with available samples (n = 282).

**Anthropometry and body composition.**

Height was measured to the nearest 0.1 cm using a portable stadiometer (Surgical and Medical PE87), and body weight to the nearest 0.01 kg with TANITA scales (bc-418, Tanita Co., Japan). Body mass index (BMI) was calculated as weight (kg) divided by height (m^2^).

**Health and medical history, and medication use.**

Participant health and medical history (past and present diagnosis of health condition from a list of 33 conditions), current medication use, ethnicity (based on birthplace and parent’s ethnic origin), education level, and smoking status, were assessed by questionnaire. Participants were deemed at cardiometabolic risk if they satisfied at least one of the following: hypertension (medication or diagnosis), lipid-lowering medication use, type 2 diabetes, history of heart attack, heart disease, or angina.

**Depression Anxiety and Stress Scale (DASS-21).**

The Depression Anxiety and Stress Scale (DASS)-21 was used to assess depression over the past week [30], with the depression subscale score used as a covariate in analyses given its association with increased risk of cognitive decline and poorer cognitive function [31].

**Habitual physical activity.**

Habitual physical activity, reported as estimated kilojoules (kJ) per week spent in moderate to high-intensity physical and leisure time activities, was evaluated using the Community Healthy Activities Model Program for Seniors (CHAMPS) questionnaire [32].

**Adverse events.**

Adverse events were defined as any unintended health-related issues (signs, symptoms, illness) that occurred or worsened during the trial. Trainers recorded any adverse events during or after exercise sessions for DT-FPT participants (e.g. illness or injury), which were categorized as ‘definitely related,’ ‘possibly related’ or ‘not related’ to the exercise intervention. Participants in both groups were also interviewed at 3, 6, 12 and 18 months by an unblinded researcher to identify and confirm any adverse events related to DT-FPT or the trial beyond usual care.

**Exercise adherence.**

Exercise adherence was tracked by monitoring attendance at supervised sessions during the initial 6-month intervention (two sessions per week) and step-down phase (one weekly session), using completed exercise cards, divided by the total prescribed sessions over 6 and 12 months and multiplied by 100.

**Statistical analyses.**

The sample size for this study was based on the primary aim of the trial which was to reduce the rate of falls [19], which after accounting for cluster randomization, yielded a sample size of 280 participants from ~15 villages. Due to higher-than-expected attrition in Cohort 1, the planned sample size was increased to 300, which provided over 80% power (2-tailed, P < 0.05) to detect modest effect sizes (e.g. Cohen’s d = 0.55) in the secondary cognitive measures of executive function and working memory, based on previous intervention effects [33, 34]. Primary statistical analyses were conducted on an intention-to-treat basis, with missing data handled on a complete case basis (valid under an assumption of data missing completely at random). Data were checked for normality before analysis. The effect of the intervention on the primary (cognition) outcomes for this study were analysed using linear mixed models with group allocation (DT-FPT or CON) as a fixed effect, while random intercepts were specified for participants nested within retirement villages to account for clustering; models were fitted with robust standard errors. The unit of analysis was the individual participant. Intervention effects on cognitive function were initially assessed by adjusting for baseline values (model 1), before adjusting for baseline values, age, sex, education, depression and BMI at baseline, cardiometabolic status, and smoking history (model 2). Within-group changes were examined using linear mixed-effects models with random intercepts, fitted separately for each group, with change from baseline as the outcome variable.

Exploratory subgroup analyses were performed to determine if genotype influenced the intervention response. Interactions between *APOE*-ε4 and *BDNF* Met-carrier status by group were conducted using linear mixed models, adjusting for all covariates in model 2 above and either *APOE*-ε4 (in BDNF analysis) or *BDNF* Met-carrier (in BDNF analysis) status. Given modest subgroup sizes, these analyses were considered hypothesis-generating. Allele and genotype frequencies were also calculated for each group, and Hardy–Weinberg equilibrium was assessed using chi-square or exact tests as appropriate. Secondary outcomes of anthropometry, body composition and habitual physical activity were analysed using linear mixed models as above, adjusted for sex and baseline values.

Additional sensitivity analyses were conducted, with multiple imputation used to handle missing data (valid under an assumption of data missing at random). Multiple imputation using chained equations was applied to impute missing data at baseline and follow-up, with 50 imputed datasets generated to ensure stability of estimates [35]. Auxiliary variables including sex, age, and cardiometabolic status were incorporated into the imputation models to improve the accuracy of imputations. Finally, per-protocol analyses were undertaken, whereby an exercise adherence of ≥80% to DT-FPT was initially prespecified, but due to the modest number meeting this level (e.g. n = 35 [22% of DT-FPT group] following the 6-month intervention, n = 26 [22% of those remaining in DT-FPT after 6-months] following the 6-month step-down period), the protocol was modified to include those with ≥50% adherence to the initial 6-month program (equivalent to one session per week), and ≥ 50% adherence to the step-down period. For completeness, per-protocol analyses were conducted based on the prespecified exercise adherence criterion of ≥80%. Given the relatively low proportion of DT-FPT participants who met these thresholds, all per-protocol results are regarded as exploratory and must be interpreted with caution.

For secondary analyses, the prevalence of mild cognitive impairment (MCI), based on CogState criteria above, was assessed at four time points (baseline, 6, 12, and 18 months). We fitted a binomial generalized linear model with a logit link to estimate the effect of group on MCI at each follow-up, adjusting for baseline MCI, with robust standard errors to account for village clustering. As a sensitivity check we also conducted generalized estimating equations (GEE) with a binomial distribution and logit link to examine the effects of group (DT-FPT vs CON), time, and their interaction on MCI prevalence, accounting for repeated measures and within-subject correlations. Robust standard errors were applied to account for clustering by village.

In addition, intraclass (intracluster) correlation coefficients (ICCs) were estimated for each cognitive outcome at each time point from two–level random-intercept models fitted by restricted maximum likelihood (REML). For most outcomes, the ICC point estimate, its standard error, and a 95% Wald confidence interval were obtained from the model’s variance components. For the remaining outcomes where the Wald interval was degenerate or unreliable (common with modest cluster counts), ICC point estimates, bootstrap standard errors, and 95% confidence intervals (CI) were obtained via 1000 cluster resampling iterations with full model refitting. Conditional ICC at baseline were calculated from random-intercept models including Group as the sole fixed effect. We used REML because it gives less biased variance-component estimates than maximum likelihood in finite samples. All statistical analyses were conducted using STATA release 17.0 (STATA, College Station, TX, USA). Descriptive data are presented as means ± SD, median and interquartile range, or counts and percentages. Statistical significance was defined as P < 0.05. No adjustments were applied for multiple testing or co-primary outcomes, to minimize the risk of over-correction and to appropriately reflect the exploratory nature of this secondary analysis. Mean estimates and 95% CI for intervention effects are reported to allow readers to assess the relative importance of findings.

## Results

**Participant characteristics.**

Three hundred men and women aged 65 to 96 years (mean ± SD; 77.4 ± 6.8) were recruited across 22 retirement communities. One DT-FPT participant withdrew after baseline, requesting data removal, hence the final number was 299 (DT-FPT, n = 155; CON, n = 144). The average size of each cluster (village) was 14 participants (range 5 to 23). Sex distribution differed between groups at baseline (DT-FPT: 65% women; CON: 82% women; Table 1), reflecting natural variation due to village-level clustering. Sex was included as a covariate for all outcome measures.

**Table 1:** Baseline characteristics of participants randomized to the dual task functional power training group (DT-FPT) and the usual care control (CON) group

Characteristics	DT-FPT	CON
n	155	144
Number of clusters (retirement communities)	11	11
Number of participants per cluster	14 (9, 17)	11 (7, 16)
Women, n (%)	101 (65%)	118 (82%)
Age, years	77.2 ± 6.6	77.7 ± 7.2
Height, cm	163.2 ± 9.1	160.0 ± 8.0
Weight, kg	77.7 ± 16.0	74.3 ± 14.1
BMI (kg/m^2^)	29.1 ± 5.2	29.0 ± 4.9
Smoking status, n (%)		
*Current/Ex-Smoker*	72 (46%)	56 (39%)
*Non-smoker*	82 (53%)	86 (60%)
DASS-21 Depression subscale score	2 (0, 6)	2 (0, 6)
*APOE*-ε4 carrier, n (%)	43 (30%)	39 (28%)
*BDNF* Met-carrier, n (%)	50 (34%)	42 (31%)
Presence of chronic health conditions*	150 (97%)	137 (96%)
*No. of conditions in those with a condition*	3 (2, 4)	3 (2, 4)
Presence of cardiometabolic risk factors, n (%)	136 (88%)	118 (82%)
Caucasian, n (%)	151 (98%)	139 (98%)
Education, n (%)		
*Primary/Some High School*	57 (37%)	57 (40%)
*Completed High School/Technical Trade Cert.*	50 (32%)	49 (34%)
*University/Tertiary level*	47 (30%)	36 (25%)
Medications taken*		
*Antihypertensive, n (%)*	112 (72%)	101 (70%)
*Lipid-lowering, n (%)*	82 (53%)	63 (44%)
*NSAIDs, n (%)*	18 (12%)	22 (15%)
*Anti-depressant, n (%)*	37 (24%)	31 (22%)
*Diabetic, n (%)*	16 (10%)	18 (13%)
*Neurological, n (%)*	4 (3%)	4 (3%)
*Analgesics (non-NSAIDs), n (%)*	17 (11%)	16 (11%)
*Sleep aids, n (%)*	11 (7%)	14 (10%)

Values are mean ± SD or n (%) except number of participants per cluster, DASS-21-Depression subscale score and number of health conditions which are reported as median (25^th^ percentile, 75^th^ percentile). APOE: apolipoprotein; BDNF: brain derived neurotrophic factor; BMI: body mass index; DASS: Depression, anxiety and stress scale. NSAID: Nonsteroidal anti-inflammatory drug. Neurological medication included anti-Parkinson and anti-epileptic medication. ^*^Missing data for DT-FPT: n = 1 (0.6%), CON: n = 2 (1.4%).

A total of 287 participants (96%) possessed at least one chronic condition at baseline, half (49%) were taking lipid-lowering medication, 71% were on antihypertensive medication, 119 (40%) were classified as obese (BMI >30; Table 1) and 43% were ex/current smokers (Table 1). Cluster-level baseline summaries for selected participant characteristics and cognitive performance variables are presented in Appendix 1. No statistically significant between-group differences were observed in medication usage changes (type, dose, or number), number of diseases (total or new), or the proportion of participants with diseases, across the study period.

At baseline, 21 (7%) of the participants were classified as having MCI, with an equal distribution between DT-FPT (n = 11, 7%) and CON (n = 10, 7%). At follow-up, of those still enrolled in the study, the prevalence of MCI was 4% (n = 5) in DT-FPT and 9% (n = 11) in CON at 6 months, 8% (n = 9) in DT-FPT and 10% (n = 11) in CON at 12 months, and 5% (n = 6) in DT-FPT and 7% (n = 8) in CON at 18 months. Across 18 months, the proportion of participants classified as MCI was generally similar between DT-FPT and CON, with only a small transient increase in CON at 6 months relative to baseline, compared to DT-FPT. Controls had higher odds of MCI at 6 months compared to baseline than the intervention group (OR 3.17, 95% CI [1.10–9.13], P = 0.033). This was confirmed by the GEE model (log-odds = 0.86 (0.06, 1.66), P = 0.035; OR = 2.36), indicating that the increase in MCI prevalence from baseline to 6 months was significantly greater in CON than in DT-FPT. No significant differences were observed at 12 or 18 months. However, given the absence of formal classification of MCI, these results are considered exploratory.

**Study Attrition.**

**Figure 1:**
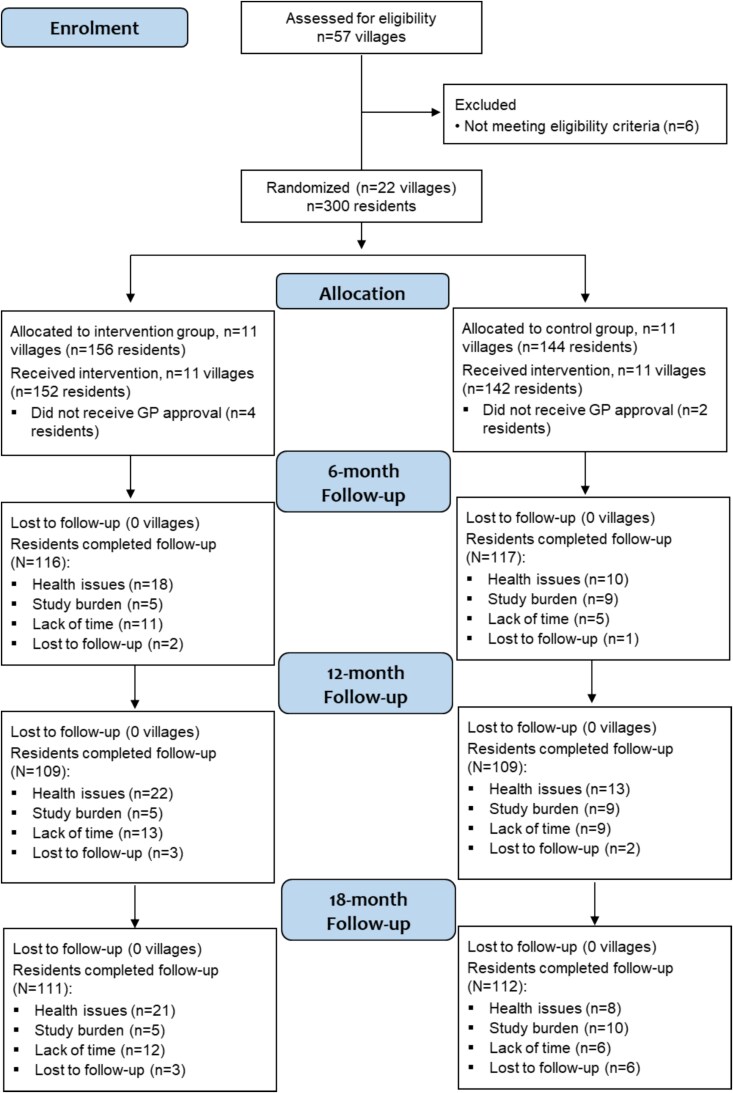
Study flow diagram of villages and participants over the 18-month study period.

In total, 67 (22%) participants (DT-FPT 25%; CON 19%) did not complete 6-month testing (Fig. 1). Of these, 49 participants withdrew (DT-FPT n = 31 [10%]; CON n = 18 [6%]), 15 could not attend to due to illness, and three were lost to follow-up. Furthermore, since there was an approximate two-week interval between baseline testing and intervention commencement for each village, six participants who were allocated to a group after baseline testing did not commence the intervention due to a subsequent lack of GP approval. By 12 months, 56 had withdrawn (DT-FPT n = 35; CON n = 21), with a further 26 unable to complete testing for health reasons (n = 15), lack of time (n = 6), or loss to follow-up (n = 5). At 18 months, 60 had withdrawn (DT-FPT n = 38; CON n = 22), while an additional 17 missed testing due to poor health (n = 6), lack of time (n = 2) or loss to follow-up (n = 9). Reasons for withdrawal or lack of follow-up are provided in Fig. 1.

**Exercise adherence and adverse events.**

Mean ± SD exercise adherence after 6 and 12 months was 50 ± 32% (median 58%, range 0 – 98%) and 40 ± 36% (median 43%, range 0 – 100%), respectively. During the 6-month intervention, 12 (8%) participants in DT-FPT reported 14 adverse events (4 related to the exercise program, 7 possibly related and 3 not related), including joint pain (knee, shoulder, back, foot), dizziness, bronchitis that was exacerbated by training, and muscle strains. Seven events resolved without issue, four persisted without treatment, and three required treatment or follow-up (e.g. icing, medication, health professional review). Trainers modified exercise programs for participants reporting an injury or complaint by reducing exercise intensity (n = 11) or temporarily discontinuing the program (n = 1 between week 17 and 18; n = 1 between week 13 and 14), while no action was required for one participant.

During the step-down phase, two minor events (feeling faint and knee pain) occurred; both were considered possibly related to the intervention and resulted in a reduction in training intensity. No participants withdrew due to injury, and all returned for follow-up testing after adverse events. When excluding DT-FPT participants who had withdrawn by the 6-month time point (n = 31), adherence among the remaining DT-FPT participants still enrolled at the beginning of the step-down phase (n = 125) was 50 ± 33% (median 60%, range 0 to 100%), based on attendance at supervised sessions.

**Cognitive Function.**

**Figure 2:**
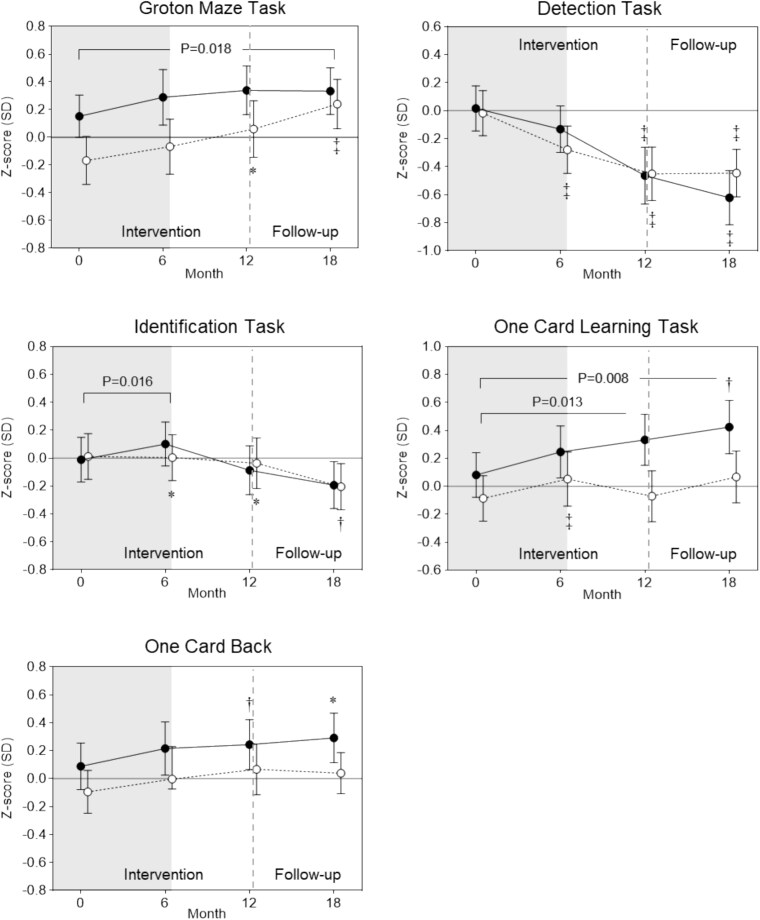
Mean (95 CI%) standardized z-scores at baseline and post each phase of the intervention (6- and 12-months) and after the follow-up period (18 months) for executive function (Groton Maze Task), psychomotor function (Detection Task), attention/choice reaction time (Identification task), visual learning (One Card Learning), and working memory (One Card Back) in the dual-task functional power training (DT-FPT) and control (CON) group. Closed dots = DT-FPT; open dots = CON. ^*^P < 0.05; † P < 0.01; ^‡^ P ≤ 0.001 within-group change relative to baseline, with significant estimated between-group differences after adjusting for age, sex, education level, cardiometabolic status, DASS-21 depression subscale score at baseline, smoking history, baseline values, and clustering, denoted by line and p-value (Model 2). Grey shaded area represents the 6-month supervised intervention period, dashed line represents end of intervention period.

Cognitive function results are shown in Table 2 and Fig. 2; both models are presented, with the focus on model 2. In the fully adjusted model (model 2), after 6 months, DT-FPT experienced an estimated mean 0.17 SD (95% CI [0.03, 0.31]) net benefit for choice reaction time/visual attention (IDN; P = 0.016) and a 0.19 SD (95% CI [0.02, 0.35]; P = 0.029) benefit in psychomotor-attention compared to CON. For the CogState Brief Battery, a 0.11 SD (95% CI [0.002, 0.22]; P = 0.046) statistically significant net benefit for DT-FPT vs CON was also found in the fully adjusted model.

**Table 2:** Mean baseline cognitive performance z-scores and adjusted within-group changes relative to baseline and net between-group differences over the 18-month intervention period in the dual-task functional power training (DT-FPT) and control (CON) groups

	**DT-FPT**			**CON**	Intervention effects	
	n	Mean ± SD or (95% CI)	N	Mean ± SD or (95% CI)	Estimated group differences (95% CI)^a^	P-values Model 1 | Model 2
Executive function (GMT)						
Baseline	151	0.15 ± 0.95	138	−0.17 ± 1.03		
∆ 6 months	113	0.01 (−0.10, 0.12)	113	0.05 (−0.08, 0.18)	0.04 (−0.15, 0.24)	0.667 | 0.531
∆ 12 months	103	0.01 (−0.15, 0.18)	106	**0.17 (0.01, 0.33)** ^*^	−0.05 (−0.30, 0.21)	0.720 | 0.660
∆ 18 months	107	−0.01 (−0.08, 0.07)	110	**0.29 (0.14, 0.44)** ^‡^	**−0.18 (−0.35, −0.02)**	**0.029** | **0.018**
**Psychomotor function (DET)**						
Baseline	154	0.02 ± 1.02	144	−0.02 ± 0.98		
∆ 6 months	115	−0.22 (−0.49, 0.05)	117	**−0.36 (−0.52, −0.21)** ^‡^	0.16 (−0.05, 0.36)	0.140 | 0.117
∆ 12 months	108	**−0.46 (−0.69, −0.24)** ^‡^	109	**−0.51 (−0.75, −0.27)** ^‡^	0.04 (−0.21, 0.30)	0.739 | 0.867
∆ 18 months	110	**−0.67 (−0.99, −0.35)** ^‡^	112	**−0.51 (−0.74, −0.28)** ^‡^	−0.17 (−0.43, 0.09)	0.207 | 0.211
**Attention/Choice reaction time (IDN)**						
Baseline	154	−0.01 ± 1.01	144	0.01 ± 1.00		
∆ 6 months	115	0.08 (−0.06, 0.22)	117	**−0.12 (−0.23, −0.005)** ^*^	**0.17 (0.04, 0.30)**	**0.009** | **0.016**
∆ 12 months	108	−0.02 (−0.17, 0.13)	109	**−0.13 (−0.24, −0.02)** ^*^	0.08 (−0.07, 0.22)	0.295 | 0.351
∆ 18 months	109	−0.18 (−0.38, 0.01)	112	**−0.24 (−0.42, −0.06)**†	0.04 (−0.15, 0.23)	0.676 | 0.691
**Visual learning (OCL)**						
Baseline	153	0.08 ± 1.00	144	−0.09 ± 0.99		
∆ 6 months	114	0.07 (−0.08, 0.23)	117	**0.20 (0.08, 0.32)** ^‡^	0.03 (−0.16, 0.22)	0.745 | 0.441
∆ 12 months	107	0.14 (−0.03, 0.30)	109	−0.02 (−0.14, 0.09)	**0.29 (0.10, 0.47)**	**0.002** | **0.013**
∆ 18 months	108	**0.22 (0.06, 0.37)**†	112	0.10 (−0.03, 0.23)	**0.22 (0.04, 0.40)**	**0.015** | **0.008**
**Working memory (ONB)**						
Baseline	154	0.09 ± 1.05	144	−0.09 ± 0.94		
∆ 6 months	115	0.07 (−0.03, 0.17)	117	0.09 (−0.03, 0.22)	0.03 (−0.12, 0.18)	0.717 | 0.664
∆ 12 months	108	**0.12 (0.03, 0.21)**†	109	0.12 (−0.02, 0.25)	0.05 (−0.13, 0.24)	0.566 | 0.898
∆ 18 months	109	**0.14 (0.004, 0.27)** ^*^	112	0.08 (−0.02, 0.18)	0.12 (−0.01, 0.26)	0.080 | 0.092
**Global cognitive function**						
Baseline	151	0.08 ± 0.69	138	−0.05 ± 0.62		
∆ 6 months	113	0.00 (−0.08, 0.08)	113	−0.03 (−0.10, 0.04)	0.08 (−0.03, 0.18)	0.148 | 0.098
∆ 12 months	103	−0.04 (−0.14, 0.07)	106	**−0.08 (−0.15, −0.01)** ^*^	0.07 (−0.04, 0.18)	0.212 | 0.229
∆ 18 months	107	−0.11 (−0.24, 0.02)	110	−0.06 (−0.16, 0.03)	−0.01 (−0.14, 0.12)	0.898 | 0.941
**Learning-Working Memory**						
Baseline	153	0.09 ± 0.83	144	−0.09 ± 0.73		
∆ 6 months	114	0.07 (−0.02, 0.16)	117	**0.14 (0.05, 0.24)**†	−0.004 (−0.13, 0.12)	0.948 | 0.761
∆ 12 months	107	**0.13 (0.04, 0.21)**†	109	0.05 (−0.04, 0.14)	**0.15 (0.02, 0.29)**	**0.030** | **0.047**
∆ 18 months	108	**0.17 (0.07, 0.28)**†	112	**0.09 (0.002, 0.18)** ^*^	**0.15 (0.01, 0.28)**	**0.029** | **0.013**
**Psychomotor function-Attention**						
Baseline	154	0.003 ± 0.89	144	−0.003 ± 0.88		
∆ 6 months	115	−0.06 (−0.24, 0.11)	117	**−0.24 (−0.36, −0.12)** ^‡^	**0.17 (0.02, 0.32)**	**0.031** | **0.029**
∆ 12 months	108	**−0.24 (−0.42, −0.06)** ^‡^	109	**−0.32 (−0.47, −0.17)** ^‡^	0.06 (−0.12, 0.25)	0.508 | 0.617
∆ 18 months	109	**−0.40 (−0.65, −0.15)**†	112	**−0.38 (−0.56, −0.19)** ^‡^	−0.05 (−0.27, 0.18)	0.683 | 0.674
**CogState Brief Battery**						
Baseline	153	0.05 ± 0.74	144	−0.05 ± 0.69		
∆ 6 months	114	0.01 (−0.09, 0.10)	117	−0.05 (−0.13, 0.04)	0.09 (−0.01, 0.19)	0.085 | **0.046**
∆ 12 months	108	−0.06 (−0.17, 0.06)	109	**−0.14 (−0.22, −0.06)** ^‡^	0.10 (−0.02, 0.22)	0.100 | 0.140
∆ 18 months	109	−0.11 (−0.27, 0.06)	112	**−0.14 (−0.25, −0.04)**†	0.05 (−0.09, 0.19)	0.499 | 0.442

Baseline values are reported as means ± SD. Within-group and estimated between-group differences are presented as means with 95% CI, adjusted for clustering. P-values for group differences were derived from linear mixed models with random intercepts for villages: Model 1 (adjusted for baseline values) and Model 2 (adjusted for age, sex, education level, cardiometabolic status, DASS-21 depression subscale score at baseline, smoking history, baseline values, and clustering). DET: Detection task; GMT: Groton Maze Learning Test; IDN: Identification task; OCL: One Card Learning task; ONB: One Back task. Bolded values indicate statistically significant within-group changes relative to baseline after adjusting for clustering, and statistically significant estimated between-group differences. ^*^P < 0.05 vs baseline; ^†^P < 0.01 vs baseline; ^‡^P ≤ 0.001 vs baseline.

^a^Estimated mean between-group differences (95% CI) were calculated from coefficients from Model 1, rather than by subtracting within-group changes from baseline for CON from within-group changes for DT-FPT at each time point.

After both 12 and 18 months, in the fully adjusted model, there was a statistically significant mean net benefit for DT-FPT compared to CON for visual learning (OCL) (0.29 SD, 95% CI [0.07, 0.51], P = 0.013; 0.27 SD, 95% CI [0.07, 0.47], P = 0.008) and learning-working memory (0.13 SD, 95% CI [0.002, 0.26], P = 0.047; 0.18 SD, 95% CI [0.04, 0.32], P = 0.013). For executive function (GMT), there was a 0.19 SD (95% CI [0.03, 0.36]) net benefit in favour of CON relative to DT-FPT after 18 months, after adjustment (P = 0.018). Intracluster correlation coefficients for each outcome are presented in Appendix 2 and were all below 0.136.

In sensitivity analyses where missing data were handled via multiple imputation, net between group differences in favour of DT-FPT were detected for psychomotor function-attention (0.18 SD, 95% CI [0.01, 0.34], P = 0.035) at 6 months, visual learning (OCL) at 12 months (0.32 SD, 95% CI [0.10, 0.54], P = 0.005) and 18 months (0.31 SD, 95% CI [0.10, 0.53], P = 0.004), and learning-working memory at 12 months (0.18 SD, 95% CI [0.03, 0.32], P = 0.016) and 18 months (0.20 SD, 95% CI [0.05, 0.35], P = 0.008) after adjusting for covariates (model 2). There was a trend for IDN at 6 months (P = 0.070), but previously reported significant effects at 6 months for the CogState Brief Battery, and the 18-month benefit for GMT in CON, were no longer statistically significant (Appendix 3).

For the 6-month per-protocol analyses (≥50% adherence after 6 months), larger benefits favouring DT-FPT were observed for choice reaction time/visual attention (IDN: 0.25 SD, 95% CI [0.11, 0.39], P < 0.001) and psychomotor-attention (0.22 SD, 95% CI [0.08, 0.36], P = 0.003; Appendix 4). There was an additional trend for a net benefit for psychomotor function in DT-FPT (DET: 0.18 SD, 95% CI [−0.001, 0.36], P = 0.051), but no statistically significant benefits for the CogState Brief Battery. There was also a benefit favouring DT-FPT for visual learning at 12 months (OCL: 0.26 SD, 95% CI [0.05, 0.47], P = 0.016) in participants with ≥50% adherence. For the 12-month per-protocol analyses (≥ 50% adherence during step-down period), there was a statistically significant net benefit for visual learning (OCL: 0.23 SD, 95% CI [0.05, 0.42], P = 0.015) in DT-FPT compared to CON at 12 months, but no benefits for learning-working memory (Appendix 5). The 6-month and 12-month per-protocol analyses (≥80% adherence after 6 months and during the step-down period) are presented in Appendices 6 and 7.

**Genotype interactions.**

Observed genotype distributions and allele frequencies are reported in Appendices 8 and 9. There was a group-by-genotype interaction for *BDNF* for learning-working memory at 18 months (0.37 SD, 95% CI [0.09, 0.66], P = 0.011) after adjusting for covariates, indicating differential treatment effects between genotypes. In Met-carriers only, there was a 0.47 SD (95% CI [0.22, 0.73]) net benefit after 18 months in DT-FPT relative to CON (P < 0.001). There was no intervention effect in Val/Val carriers (0.06 SD, 95% CI [−0.11, 0.24], P = 0.491). After adjusting for covariates, there was a group-by-genotype interaction for *BDNF* for working memory (ONB) at 18 months (0.35 SD, 95% CI [0.02, 0.68], P = 0.039). In Met-carriers, there was a 0.40 SD (95% CI [0.03, 0.78]) net benefit after 18 months in DT-FPT relative to CON (P = 0.033). There was no intervention effect in Val/Val carriers (0.01 SD, 95% CI [−0.15, 0.16], P = 0.949), and no group-by-genotype interactions for any cognitive outcome for *APOE.*

**Habitual physical activity.**

No statistically significant between group differences were detected for habitual physical activity at any time between baseline and 18 months, however there was a within-group decrease in physical activity in DT-FPT between baseline and 18 months (−1265 kJ, 95% CI [−2268, −262], P = 0.013), and a non-significant decrease in CON (−1104 kJ [95% CI: −2342, −138]; P = 0.080).

**Anthropometry and body composition.**

After adjusting for sex and baseline values, no significant between group differences were detected for anthropometry. Statistically significant within-group increases in BMI were detected in CON at 6 months (0.21 kg/m^2^, 95% CI [0.05, 0.37], P = 0.012), and DT-FPT at 18 months (0.27 kg/m^2^; 95% CI [0.06, 0.48], P = 0.013), compared to baseline.

## Discussion

The main findings from this 18-month cluster RCT of older adults at increased risk of falls residing in independent living retirement villages was that the DT-FPT intervention led to significant improvements in choice reaction time and attention, psychomotor-attention, and a composite of psychomotor function, attention, learning, and memory (CogState Brief Battery) after the 6-month supervised and structured phase. Furthermore, after 12 months of training and the 6-month follow-up, benefits from DT-FPT relative to CON were extended to attention and visual learning and learning-working memory whereas, there was a significant net benefit for executive function in CON. Furthermore, significant genotype interactions revealed benefits to working memory and the learning-working memory composite for DT-FPT versus controls in *BDNF* Met-carriers at 18 months but not in the *APOE* genotype.

The findings that 6 months of supervised and structured DT-FPT yielded benefits in choice reaction time and attention among older adults are consistent with prior meta-analyses of dual-task exercise-cognitive training, which reported improvements in cognition, including global cognition, processing speed, attention, and working memory, with effect sizes ranging from 0.2–0.5 compared to controls [5, 6, 8]. The benefits to choice reaction time and attention in our study likely stem from the multitask exercises that engaged executive abilities, particularly attention and memory, by requiring participants to divide attention across movement, visual, cognitive, and motor tasks. In line with these results, several 12-week trials including low-intensity PRT and walking [36], as well as a multicomponent intervention including PRT, aerobic, balance, coordination, and dance [37], synchronized with changing auditory cues and paired with cognitive activities like arithmetic and word games, have also reported improvements in attention in older adults. Collectively, these findings suggest that multicomponent exercise interventions incorporating dual-task activities are an effective approach to elicit improvement in specific cognitive domains.

We also found that longer term training over 12 months provided additional benefits for visual learning and learning-working memory, which may relate to participants’ continued practice of secondary cognitive tasks within training, requiring visual and verbal memory (e.g. recalling visual information, shopping lists or objects beginning with a particular letter), information manipulation (e.g. solving maths problems), information retention and retrieval, or attentional control. Furthermore, the inclusion of functional stepping tasks may decrease activation in brain areas linked to working memory (e.g. prefrontal cortex, parietal cortex, hippocampus), reflecting improved neural efficiency as motor–cognitive integration becomes more automatic [38]. Reduced cortical activation alongside stable performance reflects more efficient recruitment of sensorimotor and subcortical networks [13], supporting attention, motor control, and working memory processes. Indeed, there is some evidence that incorporating functional stepping exercises into multimodal simultaneous dual-task training can improve working memory [38, 39].

The psychomotor gains observed in our study following 6 months of DT-DPT may be attributed to visual cognitive dual-tasks requiring rapid target detection and higher-order processing (e.g. identifying differences between two images), along with motor tasks emphasizing movement synchronization with auditory or visual cues (e.g. walking to music, ball catching and throwing). Evidence suggests that functional tasks inherently involve cognitive effort, offering enriched conditions for cognitive and functional enhancements in both MCI and cognitively healthy older adults [40]. In part support, a 12-week intervention combining rapid motor tasks performed in response to audiovisual cues, concurrent task demands, and movement sequencing, integrated with moderate-intensity aerobic training and PRT (60 minutes, twice weekly), showed greater gains in simple and choice reaction time compared to exercise-only or control groups [41]. Conversely, a 12-week dual-task program focused primarily on resistance training without functional elements failed to improve reaction times [36]. Thus, we believe the benefits from our intervention may result from secondary tasks and functional training that challenged proprioception, reaction time, and information processing.

Collectively, our findings suggest that improvements in complex cognitive domains like working memory, may require more time due to intricate neurobiological adaptations. Longer exercise interventions (6–12 months) have demonstrated more consistent cognitive benefits and are associated with moderate effect sizes for cognitive benefits [42], suggesting this duration may be required to slow cognitive decline, alter disease path, and/or promote the maintenance of cognitive benefits over time [43–46]. Despite this, shorter exercise-cognitive intervention durations may still confer benefits [4, 5] by enhancing neuroplasticity and brain metabolism, with these effects reinforced by training that supports adaptive brain processes [47]. In our study, per protocol analyses indicated that participants attending ≥50% of sessions over 12 months (mean 77% adherence) demonstrated greater gains in visual learning after 12 months compared to controls. Those with ≥50% attendance (75% mean adherence) during the initial intervention phase (6-months) also showed greater benefits in choice reaction time, visual attention, and psychomotor-attention composite scores. These findings indicate that adherence is a key determinant of cognitive gains following such training, suggesting a dose–response relationship in which consistent engagement is required to elicit meaningful improvements. This is in part supported by the findings from a meta-analysis of exercise RCTs, which found that 110–170 minutes of moderate-intensity resistance and balance training per week may be required for clinically important benefits to cognition in older adults [48]. Accordingly, DT-FPT appears to be effective when adherence is maintained, underscoring the need for strategies that promote long-term engagement, such as behavioural support, social facilitation, and tailored programming, to enhance adherence and maximize real-world impact.

The finding that DT-FPT did not improve executive function or global cognition may be explained in part by the low-intensity nature of the training. Exercise-cognitive training programs including exercise sessions of a higher-intensity (above 60% of HR_max_), and aerobic or progressive resistance training, are associated with consistent improvements in global cognition and executive function in older adults [7]. In contrast, the DT-FPT sessions in our study were low-moderate intensity, focusing on challenging balance, mobility and high-velocity functional movements, with light to moderate training loads manipulated through weighted vests, therabands, and hand-held and ankle weights (0.5 to 10 kg). In conjunction with our modest adherence rates to the initial intervention (mean 50%) and step-down (mean 40%) phases which likely contributed to a lower training dose, our training intensity may have been insufficient to stimulate neurobiological mechanisms (e.g. increased neurogenesis, plasticity, hemodynamic activity) associated with exercise-induced benefits for these domains [44]. Low to moderate intensity exercise can enhance cognition through complementary mechanisms. Brain-derived neurotrophic factor-mediated neuroplasticity primarily supports memory and executive function, while cardiovascular fitness improves executive function, attention and processing speed via increased cerebral perfusion and neural efficiency [49, 50]. While our dual-task intervention was modest in intensity, it may have been sufficient to elicit improvements in domains sensitive to subtle changes, such as memory, attention, and processing speed, without reaching the threshold required to impact broader constructs such as global cognition or executive function, which may require sustained or higher-intensity stimulation [42]. In particular, improvements in neural correlates relating to higher order executive control and memory [51], may be activated by higher intensity dual-task exercise protocols.

The optimal type and dose of combined cognitive-exercise along with the underlying mechanisms for sustaining cognitive gains remain unclear, with mixed results from previous trials across follow-up periods from 3 months to 5 years [52, 53]. After 18 months, we found DT-FPT benefits in visual learning and learning-working memory persisted, with improvements in working memory also emerging. It is unclear whether these benefits are attributable to the previous 12 months of training, represent sustained effects, or reflect delayed responses. Previous studies observed improvements in divided attention after six weeks of sequential cognitive-exercise training, with greater benefits at one year follow-up [54], while global cognitive improvements were only observed 4 years after a sequential cognitive-exercise intervention [55]. It has been suggested that sustained cognitive abilities may result from increased motivation to maintain physical activity following participation in an exercise intervention [54]. During the 12 to 18-month follow-up period without training support, the DT-FPT group showed a small but statistically significant decrease of 903 kJ in weekly energy expenditure from moderate- to high-intensity physical and leisure activities. This suggests that the cognitive benefits observed are likely related to the intervention rather than maintained physical activity levels. Therefore, our findings indicate that cognitive-exercise interventions may contribute to the long-term maintenance of cognitive performance [46], although longer interventions and follow-up periods may be needed to fully evaluate their effects.

An interesting outcome from our intervention was that the effect on learning-working memory and working memory varied by genetic polymorphism, with *BDNF* Met-carriers experiencing greater benefits from DT-FPT than Val-Val carriers, but only at 18 months. Evidence on the *BDNF* Val66Met polymorphism’s interaction with exercise and cognition is limited, but it has been reported that sequential exercise-cognitive [56] and multimodal exercise training [57] can elicit cognitive benefits independent of genotype. It remains unclear whether DT-FPT influences *BDNF* gene methylation, the upregulation of circulating BDNF levels, and the consequent enhancement of cognitive function [56]. We found no differences in the intervention response by *APOE* polymorphism, supporting studies showing cognitive benefits independent of *APOE* genotype [18]. While the proportion of ε4 carriers (27%) in our study aligned with population estimates, and genotype distributions for *BDNF* and *APOE* were consistent with Hardy–Weinberg equilibrium, the modest subgroup sizes mean that false positives cannot be excluded. Larger studies are therefore needed to explore the relationship between exercise-cognitive training, genetic polymorphisms, and cognition, particularly in adults with cognitive impairment or disease [18]. These preliminary findings suggest that, in the future, genetic profiling may contribute to more individualized approaches to exercise prescription aimed at supporting cognitive health, particularly in populations at elevated risk for cognitive decline.

This study has several strengths, including being the first RCT to examine the cognitive response to a 12-month dual-task functional power-training program, which was well-tolerated with minimal adverse events. Second, cluster randomization reduced the risk of intervention contamination due to the program being conducted within each village site, and researcher blinding minimized bias. Additionally, the larger sample size, powered for the trial’s broader aims (i.e. falls), is a notable strength. However, several limitations must be considered. The modest level of adherence to DT-FPT (40–50%) likely impacted our findings but similar findings have been reported in exercise interventions in independently living older adults, highlighting the need to explore strategies that can promote adherence to such programs [58–60]. Second, a key limitation of this study is the homogeneous sample (98% Caucasian, well-educated), which, although at increased falls risk, did not meet criteria for cognitive impairment based on the validated SPMSQ. This limits generalisability to more diverse populations that may have higher baseline risk for cognitive decline, and/or frail or cognitively impaired adults who may be unable to safely undertake the DT-FPT program without individualized supervision. Future research should evaluate DT-FPT in heterogeneous cohorts to determine its broader applicability. Third, while analyses adjusted for sex and other key baseline variables, residual confounding arising from unmeasured or imperfectly measured cluster-level differences cannot be fully excluded, particularly given the cluster-randomized design, and should be considered when interpreting the cognitive outcomes. Finally, the lack of adjustment for multiple comparisons may mean that a few significant findings may be due to chance.

In conclusion, this 18-month cluster RCT underscores the potential for DT-FPT to improve certain cognitive abilities such as attention, reaction time, visual learning, working memory and psychomotor performance in older adults at increased falls risk, potentially prolonging functional independence and delaying cognitive decline. However, longer interventions with extended follow-up that incorporate inclusive strategies to promote adherence, such as behavioural support, social engagement, and flexible delivery, are needed to fully understand the long-term impact of DT-FPT on cognitive function in this population.

## Supplementary Material

aa-25-2629-File004_afaf385

aa-25-2629-File005_afaf385

aa-25-2629-File006_afaf385

aa-25-2629-File007_afaf385

aa-25-2629-File008_afaf385

aa-25-2629-File009_afaf385

aa-25-2629-File010_afaf385

aa-25-2629-File011_afaf385

aa-25-2629-File012_afaf385
