# Supplementary material for: Can dual-task high-velocity exercise training improve cognitive function in older adults? Secondary analysis of an 18-month cluster randomized controlled trial
Source: Age Ageing. 2026 Jan 23;55(1):afaf385. doi: 10.1093/ageing/afaf385 (PMC12828687; doi:10.1093/ageing/afaf385)
Supplement: aa-25-2629-File004_afaf385 [file aa-25-2629-file004_afaf385.docx]

**Appendix 1:** Cluster level baseline summaries for selected participant characteristics and cognitive performance z-scores in the dual task functional power training (DT-FPT) or usual care control (CON) groups.

| Village No. | Cluster size,n | Females,  n (%) | Age  Mean ± SD | BMI  Mean ± SD | GMT  Mean ± SD | DET  Mean ± SD | IDN  Mean ± SD | OCL  Mean ± SD | ONB  Mean ± SD | Global  Mean ± SD | L-WM  Mean ± SD | | | Psy-Att  Mean ± SD | CBB  Mean ± SD | Smoker: *Current/ Ex-Smoker* | Education *High School* | Education *University* | Cardio-metabolic risk factors |
| --- | --- | --- | --- | --- | --- | --- | --- | --- | --- | --- | --- | --- | --- | --- | --- | --- | --- | --- | --- |
| DT-FPT | | | | | | | | | | | | | | | | | | | |
| 2 | 11 | 8 (73%) | 74.4 ± 7.6 | 30.0 ± 4.5 | 0.56 ± 0.57 | 0.30 ± 0.81 | 0.11 ± 0.56 | 0.11 ± 0.99 | 0.47 ± 0.76 | 0.31 ± 0.54 | 0.29 ± 0.75 | | 0.20 ± 0.62 | | 0.25 ± 0.58 | 36% | 36% | 18% | 64% |
| 3 | 12 | 8 (67%) | 80.1 ± 7.6 | 25.4 ± 3.9 | 0.20 ± 1.15 | 0.23 ± 0.84 | -0.14 ± 0.94 | 0.06 ± 0.85 | -0.29 ± 0.92 | 0.01 ± 0.71 | -0.11 ± 0.64 | | 0.04 ± 0.84 | | -0.04 ± 0.67 | 50% | 17% | 67% | 92% |
| 4 | 11 | 7 (64%) | 79.6 ± 6.7 | 29.9 ± 5.7 | -0.59 ± 1.45 | -0.10 ± 1.16 | -0.55 ± 1.36 | -0.36 ± 1.08 | -0.40 ± 1.23 | -0.40 ± 0.80 | -0.38 ± 0.90 | | -0.32 ± 1.14 | | -0.35 ± 0.87 | 82% | 55% | 27% | 91% |
| 8 | 5 | 3 (60%) | 79.9 ± 3.5 | 28.4 ± 5.9 | -0.16 ± 0.79 | -0.75 ± 1.20 | -1.05 ± 2.17 | -0.60 ± 0.78 | -0.29 ± 1.90 | -0.29 ± 1.18 | -0.44 ± 1.23 | | -0.90 ± 1.58 | | -0.67 ± 1.36 | 40% | 40% | 0% | 100% |
| 10 | 10 | 7 (70%) | 71.3 ± 3.6 | 30.7 ± 4.0 | 0.89 ± 0.33 | 0.35 ± 0.63 | 0.46 ± 0.52 | 0.63 ± 0.81 | 0.71 ± 0.88 | 0.66 ± 0.26 | 0.78 ± 0.36 | | 0.41 ± 0.35 | | 0.61 ± 0.29 | 80% | 50% | 20% | 80% |
| 13 | 19 | 12 (63%) | 78.9 ± 6.6 | 28.9 ± 4.0 | 0.12 ± 0.67 | -0.69 ± 1.08 | -0.27 ± 0.45 | -0.15 ± 1.35 | -0.33 ± 0.83 | -0.26 ± 0.57 | -0.24 ± 0.89 | | -0.48 ± 0.65 | | -0.36 ± 0.61 | 37% | 21% | 16% | 95% |
| 14 | 21 | 14 (70%) | 75.6 ± 6.5 | 29.4 ± 4.0 | -0.23 ± 1.42 | -0.39 ± 1.17 | -0.46 ± 1.16 | -0.20 ± 0.84 | -0.06 ± 1.00 | -0.27 ± 0.60 | -0.13 ± 0.76 | | -0.42 ± 0.94 | | -0.28 ± 0.63 | 58% | 53% | 16% | 90% |
| 15 | 15 | 12 (80%) | 72.1 ± 3.2 | 30.1 ± 5.8 | 0.43 ± 0.56 | 0.36 ± 0.68 | 0.43 ± 0.88 | -0.04 ± 0.99 | 0.47 ± 0.93 | 0.33 ± 0.56 | 0.22 ± 0.81 | | 0.40 ± 0.72 | | 0.31 ± 0.70 | 47% | 13% | 13% | 93% |
| 17 | 16 | 9 (56%) | 80.6 ± 5.9 | 27.4 ± 5.0 | 0.13 ± 0.79 | 0.40 ± 1.00 | 0.24 ± 0.69 | 0.35 ± 0.65 | 0.17 ± 0.93 | 0.26 ± 0.43 | 0.26 ± 0.56 | | 0.32 ± 0.77 | | 0.29 ± 0.50 | 44% | 38% | 44% | 81% |
| 21 | 21 | 12 (57%) | 79.4 ± 5.8 | 28.7 ± 6.0 | 0.06 ± 0.77 | 0.23 ± 0.90 | 0.25 ± 1.06 | 0.34 ± 1.10 | 0.00 ± 1.32 | 0.18 ± 0.75 | 0.17 ± 1.03 | | 0.24 ± 0.90 | | 0.20 ± 0.86 | 33% | 19% | 52% | 100% |
| 22 | 15 | 9 (60%) | 76.2 ± 5.7 | 31.1 ± 7.1 | 0.47 ± 0.56 | 0.15 ± 1.00 | 0.28 ± 0.86 | 0.45 ± 0.90 | 0.52 ± 0.76 | 0.43 ± 0.40 | 0.48 ± 0.57 | | 0.21 ± 0.77 | | 0.35 ± 0.48 | 27% | 33% | 40% | 80% |
| CON | | | | | | |  |  |  |  |  | |  | |  |  |  |  |  |
| 1 | 12 | 10 (83%) | 75.3 ± 8.8 | 31.7 ± 4.9 | 0.001 ± 1.02 | 0.23 ± 0.78 | -0.08 ± 0.53 | 0.26 ± 1.30 | 0.02 ± 0.98 | 0.09 ± 0.57 | 0.14 ± 0.89 | | 0.08 ± 0.52 | | 0.11 ± 0.65 | 50% | 17% | 25% | 92% |
| 5 | 7 | 6 (86%) | 83.6 ± 3.6 | 27.2 ± 4.1 | -0.60 ± 0.61 | -0.54 ± 1.62 | 0.04 ± 1.00 | -0.83 ± 1.05 | -0.47 ± 0.55 | -0.48 ± 0.41 | -0.65 ± 0.39 | | -0.25 ± 1.20 | | -0.45 ± 0.54 | 14% | 29% | 43% | 86% |
| 6 | 8 | 6 (75%) | 84.3 ± 5.5 | 26.6 ± 2.3 | -0.20 ± 0.55 | -0.23 ± 1.28 | -0.76 ± 1.82 | 0.00 ± 0.58 | -0.69 ± 1.49 | -0.16 ± 0.70 | -0.34 ± 0.75 | | -0.49 ± 1.46 | | -0.42 ± 1.05 | 57% | 14% | 71% | 71% |
| 7 | 16 | 14 (88%) | 83.0 ± 4.8 | 27.7 ± 3.6 | -0.62 ± 1.01 | -0.54 ± 0.89 | -0.66 ± 0.86 | -0.11 ± 1.14 | -0.56 ± 1.04 | -0.53 ± 0.51 | -0.33 ± 0.64 | | -0.60 ± 0.82 | | -0.47 ± 0.49 | 33% | 33% | 27% | 80% |
| 9 | 17 | 14 (82%) | 72.5 ± 5.2 | 28.6 ± 4.9 | 0.02 ± 1.27 | 0.16 ± 0.90 | 0.23 ± 0.84 | -0.12 ± 0.66 | -0.04 ± 0.68 | 0.05 ± 0.63 | -0.08 ± 0.58 | | 0.20 ± 0.77 | | 0.06 ± 0.62 | 59% | 59% | 12% | 65% |
| 11 | 14 | 12 (86%) | 74.8 ± 6.0 | 28.3 ± 5.6 | 0.10 ± 0.91 | -0.15 ± 0.96 | 0.05 ± 1.07 | 0.01 ± 0.81 | 0.27 ± 0.66 | 0.06 ± 0.55 | 0.14 ± 0.64 | | -0.05 ± 0.80 | | 0.05 ± 0.61 | 50% | 29% | 14% | 86% |
| 12 | 23 | 17 (74%) | 73.8 ± 6.7 | 31.8 ± 5.5 | -0.21 ± 1.45 | 0.16 ± 1.02 | 0.38 ± 0.87 | -0.08 ± 0.88 | 0.19 ± 0.96 | 0.10 ± 0.70 | 0.06 ± 0.80 | | 0.27 ± 0.83 | | 0.16 ± 0.71 | 35% | 44% | 4% | 91% |
| 16 | 12 | 9 (75%) | 76.9 ± 5.5 | 30.1 ± 5.0 | 0.18 ± 0.73 | -0.03 ± 0.77 | 0.35 ± 0.40 | 0.05 ± 0.83 | 0.27 ± 0.56 | 0.16 ± 0.33 | 0.16 ± 0.57 | | 0.16 ± 0.49 | | 0.16 ± 0.35 | 42% | 17% | 50% | 92% |
| 18 | 8 | 5 (63%) | 85.0 ± 5.6 | 26.6 ± 2.2 | 0.03 ± 0.64 | 0.21 ± 0.84 | 0.26 ± 0.63 | -0.22 ± 0.74 | 0.25 ± 0.93 | 0.10 ± 0.45 | 0.01 ± 0.60 | | 0.23 ± 0.63 | | 0.12 ± 0.46 | 38% | 50% | 13% | 88% |
| 19 | 15 | 14 (93%) | 80.3 ± 6.5 | 28.4 ± 5.6 | -0.54 ± 0.96 | 0.08 ± 0.84 | -0.20 ± 0.82 | -0.39 ± 1.39 | -0.47 ± 0.70 | -0.26 ± 0.68 | -0.43 ± 0.88 | | -0.06 ± 0.76 | | -0.24 ± 0.78 | 20% | 40% | 27% | 80% |
| 20 | 12 | 11 (92%) | 76.5 ± 4.5 | 27.8 ± 4.6 | -0.08 ± 0.79 | 0.17 ± 1.09 | 0.20 ± 1.45 | 0.22 ± 1.15 | -0.16 ± 1.17 | 0.07 ± 0.73 | 0.03 ± 0.76 | | 0.19 ± 1.21 | | 0.11 ± 0.83 | 33% | 25% | 42% | 83% |
|  |  |  |  |  |  |  |  |  |  |  |  |  | | |  |  |  |  |  |

BMI: Body mass index; CBB: CogState Brief Battery; DET: Detection task; Global: Global cognition, GMT: Groton Maze Learning Test; IDN: Identification task; L-WM: Learning-working memory composite; OCL: One Card Learning task; ONB: One Back task; Psy-Att: Psychomotor function-Attention composite.
