# Supplementary material for: Can dual-task high-velocity exercise training improve cognitive function in older adults? Secondary analysis of an 18-month cluster randomized controlled trial
Source: Age Ageing. 2026 Jan 23;55(1):afaf385. doi: 10.1093/ageing/afaf385 (PMC12828687; doi:10.1093/ageing/afaf385)
Supplement: aa-25-2629-File005_afaf385 [file aa-25-2629-file005_afaf385.docx]

**Appendix 2:** Intraclass Correlations and Estimates of Between- and Within-Cluster Variance over the 18-month intervention period in the dual-task functional power training (DT-FPT) and control (CON) groups.

|  | **Intraclass correlation (Model 2)** | | | **Cluster variance** | |
| --- | --- | --- | --- | --- | --- |
|  |  | **Mean ± SE** | **95% CI** | **Between cluster variance** | **Within cluster variance** |
| **Executive function (GMT)** | | |  |  |  |
| Baseline |  | 0.038 ± 0.037 | (0.005, 0.226) | 0.061 | 0.945 |
| ∆ 6 months |  | 0.022 ± 0.046 | (0.000, 0.614) | 0.012 | 0.547 |
| ∆ 12 months |  | 0.088 ± 0.063 | (0.020, 0.310) | 0.040 | 0.417 |
| ∆ 18 months |  | 0.019 ± 0.047 | (0.000, 0.739) | 0.008 | 0.447 |
| **Psychomotor function (DET )** | | |  |  |  |
| Baseline |  | 0.054 ± 0.037 | (0.013, 0.190) | 0.049 | 0.952 |
| ∆ 6 months |  | 0.017 ± 0.037 | (0.000, 0.562) | 0.011 | 0.642 |
| ∆ 12 months |  | 0.049 ± 0.053 | (0.005, 0.328) | 0.037 | 0.717 |
| ∆ 18 months |  | 0.053 ± 0.047 | (0.009, 0.257) | 0.041 | 0.741 |
| **Attention/Choice reaction time (IDN)** | | | |  |  |
| Baseline |  | 0.085 ± 0.048 | (0.027, 0.238) | 0.078 | 0.927 |
| ∆ 6 months ^a^ |  | 0.000 ± 0.009 | (-0.017, 0.017) | 0.000 | 0.423 |
| ∆ 12 months ^a^ |  | 0.000 ± 0.018 | (-0.035, 0.035) | 0.000 | 0.381 |
| ∆ 18 months |  | 0.049 ± 0.050 | (0.006, 0.298) | 0.019 | 0.376 |
| **Visual learning (OCL)** | | |  |  |  |
| Baseline |  | 0.004 ± 0.023 | (-0.040, 0.049) | 0.008 | 0.992 |
| ∆ 6 months ^a^ |  | 0.000 ± 0.024 | (-0.048, 0.048) | 0.000 | 0.762 |
| ∆ 12 months ^a^ |  | 0.000 ± 0.020 | (-0.040, 0.040) | 0.000 | 0.727 |
| ∆ 18 months ^a^ |  | 0.000 ± 0.027 | (-0.054, 0.054) | 0.000 | 0.748 |
| **Working memory (ONB)** | | |  |  |  |
| Baseline ^a^ |  | 0.061 ± 0.042 | (0.015, 0.213) | 0.064 | 0.942 |
| ∆ 6 months |  | 0.011 ± 0.037 | (0.000, 0.885) | 0.004 | 0.351 |
| ∆ 12 months |  | 0.022 ± 0.038 | (0.001, 0.415) | 0.008 | 0.370 |
| ∆ 18 months ^a^ |  | 0.000 ± 0.023 | (-0.044, 0.044) | 0.000 | 0.362 |
| **Global cognitive function** | | |  |  |  |
| Baseline |  | 0.136 ± 0.059 | (0.055, 0.297) | 0.059 | 0.365 |
| ∆ 6 months |  | 0.043 ± 0.042 | (0.006, 0.250) | 0.006 | 0.125 |
| ∆ 12 months |  | 0.051 ± 0.050 | (0.007, 0.288) | 0.008 | 0.145 |
| ∆ 18 months |  | 0.090 ± 0.054 | (0.026, 0.264) | 0.014 | 0.139 |
| **Learning-Working Memory** | | |  |  |  |
| Baseline |  | 0.071 ± 0.046 | (0.019, 0.233) | 0.049 | 0.576 |
| ∆ 6 months ^a^ |  | 0.000 ± 0.033 | (-0.064, 0.064) | 0.000 | 0.285 |
| ∆ 12 months ^a^ |  | 0.000 ± 0.032 | (-0.063, 0.063) | 0.000 | 0.306 |
| ∆ 18 months ^a^ |  | 0.000 ± 0.033 | (-0.065, 0.065) | 0.000 | 0.289 |
| **Psychomotor function-Attention** | | |  |  |  |
| Baseline |  | 0.084 ± 0.046 | (0.028, 0.230) | 0.061 | 0.722 |
| ∆ 6 months |  | 0.005 ± 0.033 | (-0.054, 0.064) | 0.002 | 0.372 |
| ∆ 12 months |  | 0.039 ± 0.048 | (0.003, 0.338) | 0.017 | 0.408 |
| ∆ 18 months |  | 0.108 ± 0.061 | (0.034, 0.296) | 0.046 | 0.384 |
| **CogState Brief Battery** | | |  |  |  |
| Baseline |  | 0.055 ± 0.046 | (0.010, 0.249) | 0.058 | 0.457 |
| ∆ 6 months |  | 0.012 ± 0.033 | (0.000, 0.747) | 0.002 | 0.162 |
| ∆ 12 months |  | 0.022 ± 0.042 | (0.000, 0.520) | 0.005 | 0.207 |
| ∆ 18 months |  | 0.081 ± 0.054 | (0.021, 0.266) | 0.016 | 0.188 |

^a^ ICC point estimates, bootstrap standard errors, and percentile 95% confidence intervals were obtained via 1,000 cluster resampling iterations with full model refitting. Note: Intraclass correlations (ICCs) at baseline are conditional, adjusted for Group. DET: Detection task; GMT: Groton Maze Learning Test; IDN: Identification task; OCL: One Card Learning task; ONB: One Back task.
