# Supplementary material for: Can dual-task high-velocity exercise training improve cognitive function in older adults? Secondary analysis of an 18-month cluster randomized controlled trial
Source: Age Ageing. 2026 Jan 23;55(1):afaf385. doi: 10.1093/ageing/afaf385 (PMC12828687; doi:10.1093/ageing/afaf385)
Supplement: aa-25-2629-File006_afaf385 [file aa-25-2629-file006_afaf385.docx]

Appendix 3: Imputed data: Mean baseline cognitive performance z-scores, the within-group changes relative to baseline and net between-group differences over the 18-month intervention period in the dual-task functional power training (DT-FPT) and control (CON) groups.

|  | **DT-FPT** | |  | **CON** | **Intervention effects**  **s** | |
| --- | --- | --- | --- | --- | --- | --- |
|  | **n** | **Mean ± SD or**  **(95% CI)** | **N** | **Mean ± SD or**  **(95% CI)** | **Estimated group**  **Differences (95% CI) ^1^** | **P- values**  **Model 1 \| Model 2** |
| **Executive function (GMT)** | | |  |  |  |  |
| Baseline | 155 | 0.15 (0.002, 0.31) | 144 | -0.17 (-0.34, 0.003) |  |  |
| ∆ 26 weeks | 155 | 0.09 (-0.07, 0.25) | 144 | 0.07 (-0.08, 0.22) | 0.11 (-0.13, 0.35) | 0.376 \| 0.324 |
| ∆ 12 months | 155 | 0.11 (-0.07, 0.30) | 144 | **0.22 (0.06, 0.37)**† | 0.02 (-0.23, 0.27) | 0.899 \| 0.960 |
| ∆ 18 months | 155 | 0.09 (-0.03, 0.21) | 144 | **0.37 (0.21, 0.53)**‡ | -0.17 (-0.36, 0.03) | 0.094 \| 0.102 |
| **Psychomotor function (DET )** | | |  |  |  |  |
| Baseline | 155 | 0.01 (-0.15, 0.18) | 144 | -0.02 (-0.18, 0.15) |  |  |
| ∆ 26 weeks | 155 | -0.17 (-0.40, 0.05) | 144 | **-0.30 (-0.46, -0.13)**‡ | 0.17 (-0.04, 0.37) | 0.106 \| 0.066 |
| ∆ 12 months | 155 | **-0.48 (-0.67, -0.29)**‡ | 144 | **-0.46 (-0.65, -0.27)**‡ | -0.002 (-0.23, 0.22) | 0.984 \| 0.939 |
| ∆ 18 months | 155 | **-0.59 (-0.86, -0.32)**‡ | 144 | **-0.42 (-0.62, -0.23)**‡ | -0.15 (-0.38, 0.08) | 0.203 \| 0.289 |
| **Attention/Choice reaction time (IDN)** | | |  |  |  |  |
| Baseline | 155 | -0.02 (-0.18, 0.14) | 144 | 0.02 (-0.14, 0.18) |  |  |
| ∆ 26 weeks | 155 | 0.11 (-0.05, 0.27) | 144 | -0.07 (-0.23, 0.10) | 0.17 (-0.001, 0.34) | 0.050 \| 0.070 |
| ∆ 12 months | 155 | -0.03 (-0.20, 0.14) | 144 | -0.08 (-0.22, 0.05) | 0.05 (-0.10, 0.20) | 0.529 \| 0.610 |
| ∆ 18 months | 155 | **-0.19 (-0.37, -0.004)*** | 144 | -0.19 (-0.38, 0.01) | -0.002 (-0.17, 0.17) | 0.980 \| 0.177 |
| **Visual learning (OCL)** | | |  |  |  |  |
| Baseline | 155 | 0.08 (-0.08, 0.23) | 144 | -0.08 (-0.24, 0.08) |  |  |
| ∆ 26 weeks | 155 | 0.11 (-0.03, 0.26) | 144 | **0.15 (0.03, 0.27)*** | 0.04 (-0.14, 0.22) | 0.666 \| 0.433 |
| ∆ 12 months | 155 | **0.23 (0.07, 0.38)†** | 144 | -0.02 (-0.18, 0.13) | **0.34 (0.13, 0.54)** | **0.001 \| 0.005** |
| ∆ 18 months | 155 | **0.32 (0.17, 0.46)**‡ | 144 | 0.11 (-0.03, 0.26) | **0.28 (0.09, 0.48)** | **0.005 \| 0.004** |
| **Working memory (ONB)** | | |  |  |  |  |
| Baseline | 155 | 0.08 (-0.09, 0.25) | 144 | -0.09 (-0.24, 0.07) |  |  |
| ∆ 26 weeks | 155 | 0.08 (-0.03, 0.19) | 144 | **0.14 (0.002, 0.29)*** | -0.01 (0.18, 0.16) | 0.889 \| 0.846 |
| ∆ 12 months | 155 | **0.14 (0.03, 0.26)*** | 144 | 0.12 (-0.03, 0.27) | 0.07 (-0.12, 0.26) | 0.461 \| 0.610 |
| ∆ 18 months | 155 | **0.18 (0.01, 0.25)†** | 144 | **0.13 (0.02, 0.24)*** | 0.11 (-0.04, 0.27) | 0.153 \| 0.177 |
| **Global cognitive function** | | |  |  |  |  |
| Baseline | 155 | 0.06 (-0.05, 0.17) | 144 | -0.07 (-0.17, 0.04) |  |  |
| ∆ 26 weeks | 155 | 0.04 (-0.06, 0.14) | 144 | 0.001 (-0.08, 0.08) | 0.09 (-0.02, 0.20) | 0.119 \| 0.084 |
| ∆ 12 months | 155 | -0.01 (-0.12, 0.10) | 144 | -0.05 (-0.12, 0.03) | 0.08 (-0.03, 0.19) | 0.164 \| 0.226 |
| ∆ 18 months | 155 | -0.04 (-0.16, 0.08) | 144 | -0.002 (-0.10, 0.09) | 0.01 (-0.10, 0.12) | 0.899 \| 0.741 |
| **Learning-Working Memory** | | |  |  |  |  |
| Baseline | 155 | 0.08 (-0.05, 0.21) | 144 | -0.08 (-0.20, 0.04) |  |  |
| ∆ 26 weeks | 155 | 0.10 (0.01, 0.19) | 144 | **0.15 (0.05, 0.24)**† | -0.002 (-0.13, 0.12) | 0.976 \| 0.816 |
| ∆ 12 months | 155 | **0.18 (0.08, 0.29)**‡ | 144 | 0.05 (-0.05, 0.15) | **0.20 (0.05, 0.35)** | **0.008 \| 0.016** |
| ∆ 18 months | 155 | **0.25 (0.13, 0.36)**‡ | 144 | **0.12 (0.03, 0.21)**† | **0.19 (0.04, 0.33)** | **0.010 \| 0.008** |
| **Psychomotor function-Attention** | | |  |  |  |  |
| Baseline | 155 | -0.001 (-0.14, 0.14) | 144 | 0.001 (-0.14, 0.15) |  |  |
| ∆ 26 weeks | 155 | -0.03 (-0.20, 0.13) | 144 | **-0.18 (-0.33, -0.03)*** | **0.16 (0.01, 0.32)** | **0.040** \| **0.035** |
| ∆ 12 months | 155 | **-0.26 (-0.42, -0.10)**† | 144 | **-0.27 (-0.41, -0.13)**‡ | 0.02 (-0.14, 0.18) | 0.800 \| 0.857 |
| ∆ 18 months | 155 | **-0.39 (-0.59, -0.18)**‡ | 144 | **-0.31 (-0.48, -0.14)**‡ | -0.08 (-0.26, 0.10) | 0.401 \| 0.530 |
| **CogState Brief Battery** | | |  |  |  |  |
| Baseline | 155 | 0.04 (-0.08, 0.16) | 144 | -0.04 (-0.15, 0.07) |  |  |
| ∆ 26 weeks | 155 | 0.03 (-0.07, 0.14) | 144 | -0.02 (-0.11, 0.08) | 0.09 (-0.02, 0.19) | 0.119 \| 0.081 |
| ∆ 12 months | 155 | -0.04 (-0.15, 0.08) | 144 | **-0.11 (-0.20, -0.03)**† | 0.10 (-0.01, 0.22) | 0.086 \| 0.133 |
| ∆ 18 months | 155 | -0.07 (-0.21, 0.07) | 144 | -0.09 (-0.20, 0.01) | 0.05 (-0.07, 0.18) | 0.401 \| 0.335 |

Baseline values are reported as means ± SD. Within-group and estimated between-group differences are presented as means with 95% CI, adjusted for clustering. P-values for time and group-by-time interaction terms were derived from linear mixed models with random effects: Model 1 (adjusted for baseline values and clustering) and Model 2 (adjusted for age, sex, education level, cardiometabolic status, DASS-21 depression subscale score at baseline, smoking history, baseline values, and clustering). DET: Detection task; GMT: Groton Maze Learning Test; IDN: Identification task; OCL: One Card Learning task; ONB: One Back task. Bolded values indicate significant within-group changes relative to baseline after adjusting for clustering, and significant estimated between-group differences. *P<0.05 vs baseline; † P<0.01 vs baseline; ‡ P≤0.001 vs baseline.

^1^ Estimated mean between-group differences (95% CI) were calculated from coefficients from Model 1, rather than by subtracting within-group changes from baseline for CON from within-group changes for DT-FPT at each time point.
