# Supplementary material for: Can dual-task high-velocity exercise training improve cognitive function in older adults? Secondary analysis of an 18-month cluster randomized controlled trial
Source: Age Ageing. 2026 Jan 23;55(1):afaf385. doi: 10.1093/ageing/afaf385 (PMC12828687; doi:10.1093/ageing/afaf385)
Supplement: aa-25-2629-File007_afaf385 [file aa-25-2629-file007_afaf385.docx]

Appendix 4: Mean baseline cognitive performance z-scores and adjusted within-group changes relative to baseline and net between-group differences over the 18-month intervention period in the dual-task functional power training (DT-FPT) and control (CON) groups according to per protocol analyses for those with ≥50% adherence during the 6-month supervised intervention phase.

|  | **DT-FPT** | |  | **CON** | **Intervention effects**  **s** | |
| --- | --- | --- | --- | --- | --- | --- |
|  | **n** | **Mean ± SD or**  **(95% CI)** | **N** | **Mean ± SD or**  **(95% CI)** | **Estimated group**  **differences (95% CI) ^1^** | **P-values**  **Model 1 \| Model 2** |
| **Executive function (GMT)** | | |  |  |  |  |
| Baseline | 89 | 0.28 ± 0.87 | 138 | -0.17 ± 1.03 |  |  |
| ∆ 6 months | 85 | 0.06 (-0.06, 0.18) | 113 | 0.05 (-0.08, 0.18) | 0.09 (-0.13, 0.31) | 0.411 \| 0.297 |
| ∆ 12 months | 77 | 0.06 (-0.07, 0.20) | 106 | **0.17 (0.01, 0.33)*** | -0.002 (-0.24, 0.24) | 0.989 \| 0.900 |
| ∆ 18 months | 80 | 0.04 (-0.07, 0.16) | 110 | **0.29 (0.14, 0.44)**‡ | -0.14 (-0.33, 0.05) | 0.142 \| 0.171 |
| **Psychomotor function (DET )** | | |  |  |  |  |
| Baseline | 90 | 0.17 ± 0.89 | 144 | -0.02 ± 0.98 |  |  |
| ∆ 6 months | 86 | -0.25 (-0.47, -0.03)* | 117 | **-0.36 (-0.52, -0.21)**‡ | 0.15 (-0.03, 0.33) | 0.100 \| 0.051 |
| ∆ 12 months | 80 | **-0.48 (-0.71, -0.26)**‡ | 109 | **-0.51 (-0.75, -0.27)**‡ | 0.07 (-0.19, 0.34) | 0.584 \| 0.780 |
| ∆ 18 months | 82 | **-0.71 (-0.97, -0.44)**‡ | 112 | **-0.51 (-0.74, -0.28)**‡ | -0.15 (-0.43, 0.12) | 0.274 \| 0.127 |
| **Attention/Choice reaction time (IDN)** | | |  |  |  |  |
| Baseline | 90 | 0.07 ± 1.06 | 144 | 0.01 ± 1.00 |  |  |
| ∆ 6 months | 86 | 0.15 (-0.01, 0.31) | 117 | **-0.12 (-0.23, -0.005)*** | **0.24 (0.11, 0.37)** | **0.000** \| **0.000** |
| ∆ 12 months | 80 | -0.04 (-0.23, 0.15) | 109 | **-0.13 (-0.24, -0.02)*** | 0.07 (-0.10, 0.24) | 0.425 \| 0.530 |
| ∆ 18 months | 81 | -0.20 (-0.42, 0.02) | 112 | **-0.24 (-0.42, -0.06)**† | 0.03 (-0.17, 0.23) | 0.775 \| 0.871 |
| **Visual learning (OCL)** | | |  |  |  |  |
| Baseline | 90 | 0.18 ± 0.91 | 144 | -0.09 ± 0.99 |  |  |
| ∆ 6 months | 86 | -0.01 (-0.21, 0.20) | 117 | **0.20 (0.08, 0.32)**‡ | -0.04 (-0.28, 0.19) | 0.710 \| 0.922 |
| ∆ 12 months | 80 | 0.08 (-0.08, 0.24) | 109 | -0.02 (-0.14, 0.09) | **0.24 (0.06, 0.43)** | **0.010** \| **0.016** |
| ∆ 18 months | 81 | 0.15 (-0.01, 0.32) | 112 | 0.10 (-0.03, 0.23) | 0.22 (-0.05, 0.34) | 0.153 \| 0.085 |
| **Working memory (ONB)** | | |  |  |  |  |
| Baseline | 90 | 0.24 ± 1.06 | 144 | -0.09 ± 0.94 |  |  |
| ∆ 6 months | 86 | 0.04 (-0.04, 0.12) | 117 | 0.09 (-0.03, 0.22) | 0.01 (-0.12, 0.15) | 0.839 \| 0.772 |
| ∆ 12 months | 80 | 0.08 (-0.04, 0.19) | 109 | 0.12 (-0.02, 0.25) | 0.02 (-0.17, 0.22) | 0.830 \| 0.710 |
| ∆ 18 months | 81 | 0.13 (-0.01, 0.27) | 112 | 0.08 (-0.02, 0.18) | 0.13 (-0.01, 0.26) | 0.071 \| 0.101 |
| **Global cognitive function** | | |  |  |  |  |
| Baseline | 89 | 0.21 ± 0.61 | 138 | -0.05 ± 0.62 |  |  |
| ∆ 6 months | 85 | -0.01 (-0.10, 0.08) | 113 | -0.03 (-0.10, 0.04) | 0.07 (-0.04, 0.17) | 0.210 \| 0.066 |
| ∆ 12 months | 77 | -0.05 (-0.17, 0.07) | 106 | **-0.08 (-0.15, -0.01)*** | 0.05 (-0.07, 0.18) | 0.408 \| 0.327 |
| ∆ 18 months | 80 | -0.12 (-0.25, 0.0005) | 110 | -0.06 (-0.16, 0.03) | -0.03 (-0.16, 0.10) | 0.647 \| 0.805 |
| **Learning-Working Memory** | | |  |  |  |  |
| Baseline | 90 | 0.21 ± 0.78 | 144 | -0.09 ± 0.73 |  |  |
| ∆ 6 months | 86 | 0.02 (-0.11, 0.15) | 117 | **0.14 (0.05, 0.24)**† | -0.05 (-0.19, 0.08) | 0.443 \| 0.668 |
| ∆ 12 months | 80 | 0.08 (-0.03, 0.19) | 109 | 0.05 (-0.04, 0.14) | 0.11 (-0.03, 0.25) | 0.129 \| 0.103 |
| ∆ 18 months | 81 | **0.14 (0.03, 0.25)*** | 112 | **0.09 (0.002, 0.18)*** | 0.10 (-0.03, 0.24) | 0.130 \| 0.060 |
| **Psychomotor function-Attention** | | |  |  |  |  |
| Baseline | 90 | 0.12 ± 0.87 | 144 | -0.003 ± 0.88 |  |  |
| ∆ 6 months | 86 | -0.05 (-0.22, 0.12) | 117 | **-0.24 (-0.36, -0.12)**‡ | **0.19 (0.05, 0.34)** | **0.010** \| **0.003** |
| ∆ 12 months | 80 | **-0.26 (-0.46, -0.07)**† | 109 | **-0.32 (-0.47, -0.17)**‡ | 0.06 (-0.14, 0.27) | 0.536 \| 0.684 |
| ∆ 18 months | 81 | **-0.42 (-0.65, -0.19)**‡ | 112 | **-0.38 (-0.56, -0.19)**‡ | -0.05 (-0.27, 0.18) | 0.671 \| 0.490 |
| **CogState Brief Battery** | | |  |  |  |  |
| Baseline | 90 | 0.17 ± 0.72 | 144 | -0.05 ± 0.69 |  |  |
| ∆ 6 months | 86 | -0.02 (-0.13, 0.10) | 117 | -0.05 (-0.13, 0.04) | 0.07 (-0.03, 0.18) | 0.182 \| 0.062 |
| ∆ 12 months | 80 | -0.09 (-0.23, 0.04) | 109 | **-0.14 (-0.22, -0.06)**‡ | 0.08 (-0.06, 0.21) | 0.277 \| 0.296 |
| ∆ 18 months | 81 | -0.14 (-0.29, 0.01) | 112 | **-0.14 (-0.25, -0.04)**† | 0.02 (-0.12, 0.16) | 0.736 \| 0.668 |

Baseline values are reported as means ± SD. Within-group and estimated between-group differences are presented as means with 95% CI, adjusted for clustering. P-values for group differences were derived from linear mixed models with random intercepts for villages: Model 1 (adjusted for baseline values) and Model 2 (adjusted for age, sex, education level, cardiometabolic status, DASS-21 depression subscale score at baseline, smoking history, and baseline values). DET: Detection task; GMT: Groton Maze Learning Test; IDN: Identification task; OCL: One Card Learning task; ONB: One Back task. Bolded values indicate statistically significant within-group changes relative to baseline after adjusting for clustering and statistically significant estimated between-group differences. *P<0.05 vs baseline; † P<0.01 vs baseline; ‡ P≤0.001 vs baseline.

^1^ Estimated mean between-group differences (95% CI) were calculated from coefficients from Model 1, rather than by subtracting within-group changes from baseline for CON from within-group changes for DT-FPT at each time point.
