# Supplementary material for: Can dual-task high-velocity exercise training improve cognitive function in older adults? Secondary analysis of an 18-month cluster randomized controlled trial
Source: Age Ageing. 2026 Jan 23;55(1):afaf385. doi: 10.1093/ageing/afaf385 (PMC12828687; doi:10.1093/ageing/afaf385)
Supplement: aa-25-2629-File008_afaf385 [file aa-25-2629-file008_afaf385.docx]

Appendix 5: Mean baseline cognitive performance z-scores and adjusted within-group changes relative to baseline and net between-group differences over the 18-month intervention period in the dual-task functional power training (DT-FPT) and control (CON) groups according to per protocol analyses for those with ≥50% adherence during the step-down intervention phase (between 6 and 12 months).

|  | **DT-FPT** | |  | **CON** | **Intervention effects**  **s** | |
| --- | --- | --- | --- | --- | --- | --- |
|  | **n** | **Mean ± SD or**  **(95% CI)** | **N** | **Mean ± SD or**  **(95% CI)** | **Estimated group**  **differences (95% CI) ^1^** | **P-values**  **Model 1 \| Model 2** |
| **Executive function (GMT)** | | |  |  |  |  |
| Baseline | 76 | 0.29 ± 0.91 | 138 | -0.17 ± 1.03 |  |  |
| ∆ 6 months | — | — | — | — | — | — |
| ∆ 12 months | 72 | 0.08 (-0.07, 0.23) | 106 | **0.17 (0.01, 0.33)*** | 0.01 (-0.23, 0.26) | 0.909 \| 0.945 |
| ∆ 18 months | 72 | 0.05 (-0.07, 0.16) | 110 | **0.29 (0.14, 0.44)**‡ | -0.14 (-0.34, 0.06) | 0.162 \| 0.180 |
| **Psychomotor function (DET )** | | |  |  |  |  |
| Baseline | 77 | 0.11 ± 0.89 | 144 | -0.02 ± 0.98 |  |  |
| ∆ 6 months | — | — | — | — | — | — |
| ∆ 12 months | 74 | **-0.39 (-0.58, -0.20)**‡ | 109 | **-0.51 (-0.75, -0.27)**‡ | 0.15 (-0.11, 0.41) | 0.251 \| 0.493 |
| ∆ 18 months | 74 | **-0.65 (-0.87, -0.43)**‡ | 112 | **-0.51 (-0.74, -0.28)**‡ | -0.12 (-0.38, 0.14) | 0.363 \| 0.111 |
| **Attention/Choice reaction time (IDN)** | | |  |  |  |  |
| Baseline | 77 | 0.03 ± 1.11 | 144 | 0.01 ± 1.00 |  |  |
| ∆ 6 months | — | — | — | — | — | — |
| ∆ 12 months | 74 | 0.001 (-0.19, 0.20) | 109 | **-0.13 (-0.24, -0.02)*** | 0.11 (-0.06, 0.28) | 0.222 \| 0.334 |
| ∆ 18 months | 73 | -0.16 (-0.39, 0.08) | 112 | **-0.24 (-0.42, -0.06)**† | 0.06 (-0.14, 0.27) | 0.542 \| 0.728 |
| **Visual learning (OCL)** | | |  |  |  |  |
| Baseline | 77 | 0.21 ± 0.91 | 144 | -0.09 ± 0.99 |  |  |
| ∆ 6 months | — | — | — | — | — | — |
| ∆ 12 months | 74 | 0.06 (-0.09, 0.21) | 109 | -0.02 (-0.14, 0.09) | **0.23 (0.06, 0.40)** | **0.007** \| **0.015** |
| ∆ 18 months | 73 | 0.15 (-0.04, 0.34) | 112 | 0.10 (-0.03, 0.23) | 0.16 (-0.03, 0.36) | 0.104 \| 0.111 |
| **Working memory (ONB)** | | |  |  |  |  |
| Baseline | 77 | 0.27 ± 1.08 | 144 | -0.09 ± 0.94 |  |  |
| ∆ 6 months | — | — | — | — | — | — |
| ∆ 12 months | 74 | 0.09 (-0.01, 0.19) | 109 | 0.12 (-0.02, 0.25) | 0.04 (-0.15, 0.24) | 0.675 \| 0.834 |
| ∆ 18 months | 73 | 0.12 (-0.04, 0.28) | 112 | 0.08 (-0.02, 0.18) | 0.14 (-0.01, 0.30) | 0.076 \| 0.137 |
| **Global cognitive function** | | |  |  |  |  |
| Baseline | 76 | 0.21 ± 0.63 | 138 | -0.05 ± 0.62 |  |  |
| ∆ 6 months | — | — | — | — | — | — |
| ∆ 12 months | 72 | -0.03 (-0.15, 0.08) | 106 | **-0.08 (-0.15, -0.01)*** | 0.08 (-0.04, 0.20) | 0.211 \| 0.217 |
| ∆ 18 months | 72 | -0.11 (-0.22, 0.01) | 110 | -0.06 (-0.16, 0.03) | -0.01 (-0.13, 0.12) | 0.903 \| 0.919 |
| **Learning-Working Memory** | | |  |  |  |  |
| Baseline | 77 | 0.24 ± 0.79 | 144 | -0.09 ± 0.73 |  |  |
| ∆ 6 months | — | — | — | — | — | — |
| ∆ 12 months | 74 | 0.08 (-0.03, 0.18) | 109 | 0.05 (-0.04, 0.14) | 0.11 (-0.02, 0.25) | 0.114 \| 0.101 |
| ∆ 18 months | 73 | **0.14 (0.02, 0.25)*** | 112 | **0.09 (0.002, 0.18)*** | 0.12 (-0.02, 0.26) | 0.101 \| 0.074 |
| **Psychomotor function-Attention** | | |  |  |  |  |
| Baseline | 77 | 0.07 ± 0.89 | 144 | -0.003 ± 0.88 |  |  |
| ∆ 6 months | — | — | — | — | — | — |
| ∆ 12 months | 74 | **-0.20 (-0.38, -0.02)*** | 109 | **-0.32 (-0.47, -0.17)**‡ | 0.12 (-0.07, 0.32) | 0.213 \| 0.375 |
| ∆ 18 months | 73 | **-0.37 (-0.59, -0.16)**‡ | 112 | **-0.38 (-0.56, -0.19)**‡ | -0.01 (-0.22, 0.21) | 0.946 \| 0.633 |
| **CogState Brief Battery** | | |  |  |  |  |
| Baseline | 77 | 0.15 ± 0.73 | 144 | -0.05 ± 0.69 |  |  |
| ∆ 6 months | — | — | — | — | — | — |
| ∆ 12 months | 74 | -0.06 (-0.19, 0.06) | 109 | **-0.14 (-0.22, -0.06)**‡ | 0.11 (-0.02, 0.24) | 0.093 \| 0.112 |
| ∆ 18 months | 73 | -0.12 (-0.27, 0.03) | 112 | **-0.14 (-0.25, -0.04)**† | 0.05 (-0.09, 0.19) | 0.461 \| 0.560 |

Baseline values are reported as means ± SD. Within-group and estimated between-group differences are presented as means with 95% CI, adjusted for clustering. P-values for group differences were derived from linear mixed models with random intercepts for villages: Model 1 (adjusted for baseline values) and Model 2 (adjusted for age, sex, education level, cardiometabolic status, DASS-21 depression subscale score at baseline, smoking history, baseline values, and clustering). DET: Detection task; GMT: Groton Maze Learning Test; IDN: Identification task; OCL: One Card Learning task; ONB: One Back task. Bolded values indicate statistically significant within-group changes relative to baseline after adjusting for clustering, and statistically significant estimated between-group differences. *P<0.05 vs baseline; † P<0.01 vs baseline; ‡ P≤0.001 vs baseline.

^1^ Estimated mean between-group differences (95% CI) were calculated from coefficients from Model 1, rather than by subtracting within-group changes from baseline for CON from within-group changes for DT-FPT at each time point. NB: As analysis is based on adherence during the step-down phase, results from 6-months were not retrospectively analysed or included.
