# Supplementary material for: Can dual-task high-velocity exercise training improve cognitive function in older adults? Secondary analysis of an 18-month cluster randomized controlled trial
Source: Age Ageing. 2026 Jan 23;55(1):afaf385. doi: 10.1093/ageing/afaf385 (PMC12828687; doi:10.1093/ageing/afaf385)
Supplement: aa-25-2629-File009_afaf385 [file aa-25-2629-file009_afaf385.docx]

Appendix 6: Mean baseline cognitive performance z-scores and adjusted within-group changes relative to baseline and net between-group differences over the 18-month intervention period in the dual-task functional power training (DT-FPT) and control (CON) groups according to per protocol analyses for those with ≥80% adherence during the 6-month supervised intervention phase.

|  | **DT-FPT** | |  | **CON** | **Intervention effects**  **s** | |
| --- | --- | --- | --- | --- | --- | --- |
|  | **n** | **Mean ± SD or**  **(95% CI)** | **N** | **Mean ± SD or**  **(95% CI)** | **Estimated group**  **differences (95% CI) ^1^** | **P-values**  **Model 1 \| Model 2** |
| **Executive function (GMT)** | | |  |  |  |  |
| Baseline | 35 | 0.19 ± 1.03 | 138 | -0.17 ± 1.03 |  |  |
| ∆ 6 months | 34 | 0.07 (-0.14, 0.28) | 113 | 0.05 (-0.08, 0.18) | 0.09 (-0.27, 0.45) | 0.620 \| 0.429 |
| ∆ 12 months | 32 | 0.14 (-0.05, 0.34) | 106 | **0.17 (0.01, 0.33)*** | 0.08 (-0.16, 0.32) | 0.517 \| 0.552 |
| ∆ 18 months | 33 | 0.18 (-0.03, 0.39) | 110 | **0.29 (0.14, 0.44)**‡ | 0.003 (-0.20, 0.21) | 0.974 \| 0.867 |
| **Psychomotor function (DET )** | | |  |  |  |  |
| Baseline | 35 | 0.24 ± 0.81 | 144 | -0.02 ± 0.98 |  |  |
| ∆ 6 months | 34 | **-0.27 (-0.50, -0.05)*** | 117 | **-0.36 (-0.52, -0.21)**‡ | 0.15 (-0.07, 0.36) | 0.185 \| **0.016** |
| ∆ 12 months | 33 | **-0.54 (-0.84, -0.25)**‡ | 109 | **-0.51 (-0.75, -0.27)**‡ | 0.03 (-0.33, 0.38) | 0.884 \| 0.791 |
| ∆ 18 months | 34 | **-0.98 (-1.24, -0.73)**‡ | 112 | **-0.51 (-0.74, -0.28)**‡ | **-0.40 (-0.71, -0.10)** | **0.009 \| 0.001** |
| **Attention/Choice reaction time (IDN)** | | |  |  |  |  |
| Baseline | 35 | -0.001 ± 0.71 | 144 | 0.01 ± 1.00 |  |  |
| ∆ 6 months | 34 | 0.17 (-0.04, 0.38) | 117 | **-0.12 (-0.23, -0.005)*** | **0.22 (0.06, 0.38)** | **0.006** \| **0.017** |
| ∆ 12 months | 33 | -0.09 (-0.25, 0.08) | 109 | **-0.13 (-0.24, -0.02)*** | 0.01 (-0.14, 0.17) | 0.873 \| 0.908 |
| ∆ 18 months | 34 | **-0.32 (-0.52, -0.12)†** | 112 | **-0.24 (-0.42, -0.06)**† | -0.09 (-0.31, 0.13) | 0.435 \| 0.327 |
| **Visual learning (OCL)** | | |  |  |  |  |
| Baseline | 35 | 0.38 ± 0.93 | 144 | -0.09 ± 0.99 |  |  |
| ∆ 6 months | 34 | -0.09 (-0.30, 0.12) | 117 | **0.20 (0.08, 0.32)**‡ | -0.14 (-0.37, 0.10) | 0.253 \| 0.450 |
| ∆ 12 months | 33 | 0.15 (-0.07, 0.37) | 109 | -0.02 (-0.14, 0.09) | **0.28 (0.06, 0.51)** | **0.014** \| **0.002** |
| ∆ 18 months | 33 | **0.20 (0.03, 0.37)*** | 112 | 0.10 (-0.03, 0.23) | 0.18 (-0.01, 0.38) | 0.068 \| 0.056 |
| **Working memory (ONB)** | | |  |  |  |  |
| Baseline | 35 | 0.38 ± 0.93 | 144 | -0.09 ± 0.94 |  |  |
| ∆ 6 months | 34 | -0.02 (-0.15, 0.11) | 117 | 0.09 (-0.03, 0.22) | -0.03 (-0.22, 0.17) | 0.803 \| 0.694 |
| ∆ 12 months | 33 | **-**0.03 (-0.21, 0.15) | 109 | 0.12 (-0.02, 0.25) | -0.04 (-0.27, 0.18) | 0.703 \| 0.367 |
| ∆ 18 months | 33 | 0.05 (-0.20, 0.31) | 112 | 0.08 (-0.02, 0.18) | 0.11 (-0.12, 0.35) | 0.339 \| 0.462 |
| **Global cognitive function** | | |  |  |  |  |
| Baseline | 35 | 0.20 ± 0.56 | 138 | -0.05 ± 0.62 |  |  |
| ∆ 6 months | 34 | -0.03 (-0.13, 0.07) | 113 | -0.03 (-0.10, 0.04) | 0.04 (-0.08, 0.16) | 0.522 \| 0.138 |
| ∆ 12 months | 32 | -0.04 (-0.18, 0.09) | 106 | **-0.08 (-0.15, -0.01)*** | 0.06 (-0.06, 0.18) | 0.349 \| 0.179 |
| ∆ 18 months | 80 | **-0.16 (-0.27, -0.05)†** | 110 | -0.06 (-0.16, 0.03) | -0.06 (-0.17, 0.06) | 0.336 \| 0.533 |
| **Learning-Working Memory** | | |  |  |  |  |
| Baseline | 35 | 0.28 ± 0.75 | 144 | -0.09 ± 0.73 |  |  |
| ∆ 6 months | 34 | -0.06 (-0.14, 0.03) | 117 | **0.14 (0.05, 0.24)**† | **-0.12 (-0.23, -0.01)** | **0.037** \| 0.108 |
| ∆ 12 months | 33 | 0.06 (-0.10, 0.22) | 109 | 0.05 (-0.04, 0.14) | 0.11 (-0.07, 0.30) | 0.229 \| 0.108 |
| ∆ 18 months | 33 | **0.12 (0.002, 0.24)*** | 112 | **0.09 (0.002, 0.18)*** | 0.12 (-0.01, 0.25) | 0.067 \| 0.076 |
| **Psychomotor function-Attention** | | |  |  |  |  |
| Baseline | 35 | 0.12 ± 0.69 | 144 | -0.003 ± 0.88 |  |  |
| ∆ 6 months | 34 | -0.05 (-0.24, 0.14) | 117 | **-0.24 (-0.36, -0.12)**‡ | **0.18 (0.01, 0.35)** | **0.036** \| **0.003** |
| ∆ 12 months | 33 | **-0.31 (-0.51, -0.10)**† | 109 | **-0.32 (-0.47, -0.17)**‡ | 0.02 (-0.21, 0.25) | 0.856 \| 0.784 |
| ∆ 18 months | 33 | **-0.61 (-0.79, -0.43)**‡ | 112 | **-0.38 (-0.56, -0.19)**‡ | **-0.22 (-0.43, -0.005)** | **0.045** **\| 0.024** |
| **CogState Brief Battery** | | |  |  |  |  |
| Baseline | 35 | 0.20 ± 0.57 | 144 | -0.05 ± 0.69 |  |  |
| ∆ 6 months | 34 | -0.05 (-0.17, 0.06) | 117 | -0.05 (-0.13, 0.04) | 0.03 (-0.07, 0.13) | 0.557 \| 0.109 |
| ∆ 12 months | 33 | -0.12 (-0.29, 0.04) | 109 | **-0.14 (-0.22, -0.06)**‡ | 0.05 (-0.12, 0.21) | 0.576 \| 0.364 |
| ∆ 18 months | 33 | **-0.24 (-0.37, -0.12)‡** | 112 | **-0.14 (-0.25, -0.04)**† | -0.06 (-0.19, 0.07) | 0.359 \| 0.428 |

Baseline values are reported as means ± SD. Within-group and estimated between-group differences are presented as means with 95% CI, adjusted for clustering. P-values for group differences were derived from linear mixed models with random intercepts for villages: Model 1 (adjusted for baseline values) and Model 2 (adjusted for age, sex, education level, cardiometabolic status, DASS-21 depression subscale score at baseline, smoking history, baseline values, and clustering). DET: Detection task; GMT: Groton Maze Learning Test; IDN: Identification task; OCL: One Card Learning task; ONB: One Back task. Bolded values indicate statistically significant within-group changes relative to baseline after adjusting for clustering, and statistically significant estimated between-group differences. *P<0.05 vs baseline; † P<0.01 vs baseline; ‡ P≤0.001 vs baseline.

^1^ Estimated mean between-group differences (95% CI) were calculated from coefficients from Model 1, rather than by subtracting within-group changes from baseline for CON from within-group changes for DT-FPT at each time point.
