# Supplementary material for: Can dual-task high-velocity exercise training improve cognitive function in older adults? Secondary analysis of an 18-month cluster randomized controlled trial
Source: Age Ageing. 2026 Jan 23;55(1):afaf385. doi: 10.1093/ageing/afaf385 (PMC12828687; doi:10.1093/ageing/afaf385)
Supplement: aa-25-2629-File011_afaf385 [file aa-25-2629-file011_afaf385.docx]

**Appendix 8:** Group by *APOE* and *BDNF* genotype by time cell sizes over the 18-month intervention period in the dual-task functional power training (DT-FPT) and control (CON) groups.

|  | **Baseline, n (%)** | **6 months, n (%)** | **12 months, n (%)** | **18 months, n (%)** |
| --- | --- | --- | --- | --- |
| *APOE* **Genotype** |  |  |  |  |
| **DT-FPT** |  |  |  |  |
| ε2/ε2 | 1 (1%) | 1 (1%) | 1 (1%) | 1 (1%) |
| ε2/ε3 | 16 (11%) | 12 (11%) | 12 (11%) | 12 (11%) |
| ε2/ε4 | 3 (2%) | 1 (1%) | 1 (1%) | 1 (1%) |
| ε3/ε3 | 85 (59%) | 68 (60%) | 63 (59%) | 66 (1%) |
| ε3/ε4 | 36 (25%) | 27 (24%) | 25 (24%) | 24 (22%) |
| ε4/ε4 | 4 (3%) | 4 (4%) | 4 (4%) | 4 (4%) |
| **Total n** | **145** | **113** | **106** | **108** |
| **CON** |  |  |  |  |
| ε2/ε2 | 0 (0%) | 0 (0%) | 0 (0%) | 0 (0%) |
| ε2/ε3 | 9 (7%) | 7 (6%) | 6 (6%) | 6 (5%) |
| ε2/ε4 | 4 (3%) | 3 (3%) | 3 (3%) | 3 (3%) |
| ε3/ε3 | 89 (65%) | 78 (68%) | 69 (64%) | 73 (66%) |
| ε3/ε4 | 33 (24%) | 24 (21%) | 27 (25%) | 27 (25%) |
| ε4/ε4 | 2 (1%) | 2 (2%) | 2 (2%) | 1 (1%) |
| **Total n** | **137** | **114** | **107** | **110** |
|  |  |  |  |  |
| ***BDNF* Genotype** |  |  |  |  |
| **DT-FPT** |  |  |  |  |
| Val66Val | 95 (66%) | 76 (67%) | 71 (67%) | 72 (67%) |
| Val66Met | 45 (31%) | 33 (29%) | 31 (29%) | 32 (30%) |
| Met66Met | 5 (3%) | 4 (4%) | 4 (4%) | 4 (4%) |
| **Total n** | **145** | **113** | **106** | **108** |
| **CON** |  |  |  |  |
| Val66Val | 95 (69%) | 80 (70%) | 74 (69%) | 77 (70%) |
| Val66Met | 38 (28%) | 30 (26%) | 30 (28%) | 30 (27%) |
| Met66Met | 4 (3%) | 4 (4%) | 3 (3%) | 3 (3%) |
| **Total n** | **137** | **114** | **107** | **110** |
| **Sample total** | **282** | **227** | **213** | **218** |

NB: Counts and percentages represent participants who attended functional testing and provided cognitive data at the respective time points. Genotype data was provided by n=282 participants.
