# Supplementary material for: Can dual-task high-velocity exercise training improve cognitive function in older adults? Secondary analysis of an 18-month cluster randomized controlled trial
Source: Age Ageing. 2026 Jan 23;55(1):afaf385. doi: 10.1093/ageing/afaf385 (PMC12828687; doi:10.1093/ageing/afaf385)
Supplement: aa-25-2629-File012_afaf385 [file aa-25-2629-file012_afaf385.docx]

**Appendix 9.** Hardy-Weinberg Equilibrium analysis of *APOE* and *BDNF* genotypes in the dual-task functional power training (DT-FPT) and control (CON) groups.

|  | **DT-FPT Observed, n (%)** | **DT-FPT Expected, n** | **CON**  **Observed, n (%)** | **CON**  **Expected, n** |
| --- | --- | --- | --- | --- |
| ***APOE* Genotype** |  |  |  |  |
| ε2/ε2 | 1 (1%) | 0.76 | 0 (0%) | 0.31 |
| ε2/ε3 | 16 (11%) | 16.08 | 9 (7%) | 10.44 |
| ε2/ε4 | 3 (2%) | 3.40 | 4 (3%) | 1.95 |
| ε3/ε3 | 85 (59%) | 84.97 | 89 (65%) | 88.32 |
| ε3/ε4 | 36 (25%) | 35.98 | 33 (24%) | 32.92 |
| ε4/ε4 | 4 (3%) | 3.81 | 2 (1%) | 3.07 |
| **Total n** | **145** |  | **137** |  |
| Exact p-value^1^ | 0.902 |  | 0.457 |  |
| χ² (df=5)^2^ | 0.133 |  | 3.054 |  |
| p-value^2^ | 1.000 |  | 0.691 |  |
|  |  |  |  |  |
| ***BDNF* Genotype** |  |  |  |  |
| Val66Val | 95 (66%) | 95.3 | 95 (69%) | 94.9 |
| Val66Met | 45 (31%) | 44.6 | 38 (28%) | 38.3 |
| Met66Met | 5 (3%) | 5.0 | 4 (3%) | 3.8 |
| **Total n** | **145** |  | **137** |  |
| Exact p-value^1^ | 1.000 |  | 1.000 |  |
| χ² (df=1)^2^ | 0.007 |  | 0.014 |  |
| p-value^2^ | 0.933 |  | 0.906 |  |

^1^All genotypes were included in analysis; exact test applied to account for sparse categories. Mid‑p values are reported.

^2^Chi‑squared test applied across all genotypes; note that expected counts <5 in some categories may limit the validity of the approximation.

Genotype data was provided by n=282 participants.
